# Targeting cancer-intrinsic protein neddylation bypasses the loss of JAK/STAT signaling and overcomes acquired resistance to immunotherapy

**DOI:** 10.21203/rs.3.rs-9107846/v1

**Published:** 2026-08-06

**Authors:** Yumeng Mao, Marta Rubies Bedos, Yonglin Lu, Eirini Voutsinou, Irineos Papakyriacou, Liam Alford, Rebeca Parracho, Vaishnavi Iyer, Antoni Ribas

**Affiliations:** Uppsala University; Uppsala University; Uppsala University; Uppsala University; Uppsala University; Uppsala University; Uppsala University; Uppsala University; U.C.L.A.

## Abstract

The loss of IFNγ signaling in cancer cells causes primary and acquired resistance to immunotherapy. Using a patient-derived JAK2-deficient melanoma cell line, we have developed a co-culture system to model acquired resistance to immunotherapy. Genome-wide genetic screens uncover that protein neddylation can be targeted to restore sensitivity to PD1 blockade in co-cultures, and in MHC-I and/or JAK/STAT-deficient murine tumors. Analysis of the Jak2-deficient murine tumors with scRNA-seq reveals a heightened cancer-intrinsic inflammatory signature when neddylation is absent, which causes an influx of stimulatory antigen presenting cells. In patients with melanoma, tumors with low cancer-intrinsic neddylation exhibit elevated IFN signatures and are associated with dendritic cell infiltration. Mechanistically, neddylation deletion stabilizes chromatin-bound cGAS in JAK2-deficient cancer cells, which is responsible for the superior response to PD1 blockade, and the recruitment of pro-inflammatory innate immune cells. Altogether, we demonstrate the broad therapeutic potential of protein neddylation in overcoming resistance to immunotherapy.

## Introduction

Immune checkpoint blockade (ICB) therapies reinvigorate the immune system to eliminate cancer cells and have been approved in several tumor types. However, multiple mechanisms in the tumor microenvironment hamper efficient anti-tumor immunity [1] and contribute to therapy resistance [2]. In particular, loss of the β2-microglobulin (*B2M*) gene in cancer cells destabilizes the antigen presentation machinery to CD8+ T cells [3], which is a well-recognized immune evasion mechanism in cancer development and immunotherapy [4–7]. Moreover, deficiency of Janus Kinases 1/2 (JAK1/2) in cancer cells leads to primary and acquired resistance to ICB therapy in melanoma and mismatch repair-deficient colon cancer patients [4, 8–11]. In accordance, deletion of the *B2m* gene [6, 7, 12], the *Jak1/2* gene [13–15] or the interferon receptors [10] in murine cancer cells confers resistance to ICB therapy in syngeneic mouse models, partly due to the failure to upregulate PD-L1 in cancer cells [14, 16]. Simultaneous activation of myeloid and NK cells [13] or intra-tumoral delivery of agents that turn on IFN-stimulated genes [13, 15, 17] overcomes acquired resistance in tumor-bearing mice.

Genome-wide CRISPR/Cas9 screens in syngeneic mouse models have been employed to unravel cancer-intrinsic resistance mechanisms to ICB [18, 19] and validated the therapeutic potential of *PTPN2* as a negative regulator for the JAK/STAT signaling in cancer cells [18, 20]. Moreover, we have optimized a workflow to uncover and validate cancer vulnerabilities to clinically approved ICB drugs using genome-wide CRISPR screens in a human Tumor-Immune co-Culture System (TICS) [21, 22]. To our knowledge, unbiased and genome-wide genetic screens are yet to be applied to uncover mechanisms that can revert acquired resistance to ICB therapy due to the loss of JAK/STAT signaling pathway.

In this study, we performed genome-wide CRISPR screens in TICS using a melanoma cell line (M464), which was established from a patient with acquired resistance to pembrolizumab due to a loss-of-function mutation on the *JAK2* gene [4]. This approach identified protein neddylation as a target to overcome acquired resistance to ICB. In mice, neddylation-deficient tumors overcame acquired resistance to PD1 blockade in tumors lacking the *B2m* and/or *Jak1/2* genes. Single-cell transcriptomics analysis of murine or patient tumors, as well as mechanistic studies using genetic deletions, pinpointed the essential role of protein neddylation in regulating chromatin-bound cGAS, which was found to activate multiple cell types of the innate immune system.

## Results

### Genome-wide CRISPR/Cas9 screens identify targets to overcome acquired resistance to PD1 blockade

**Figure 1:**
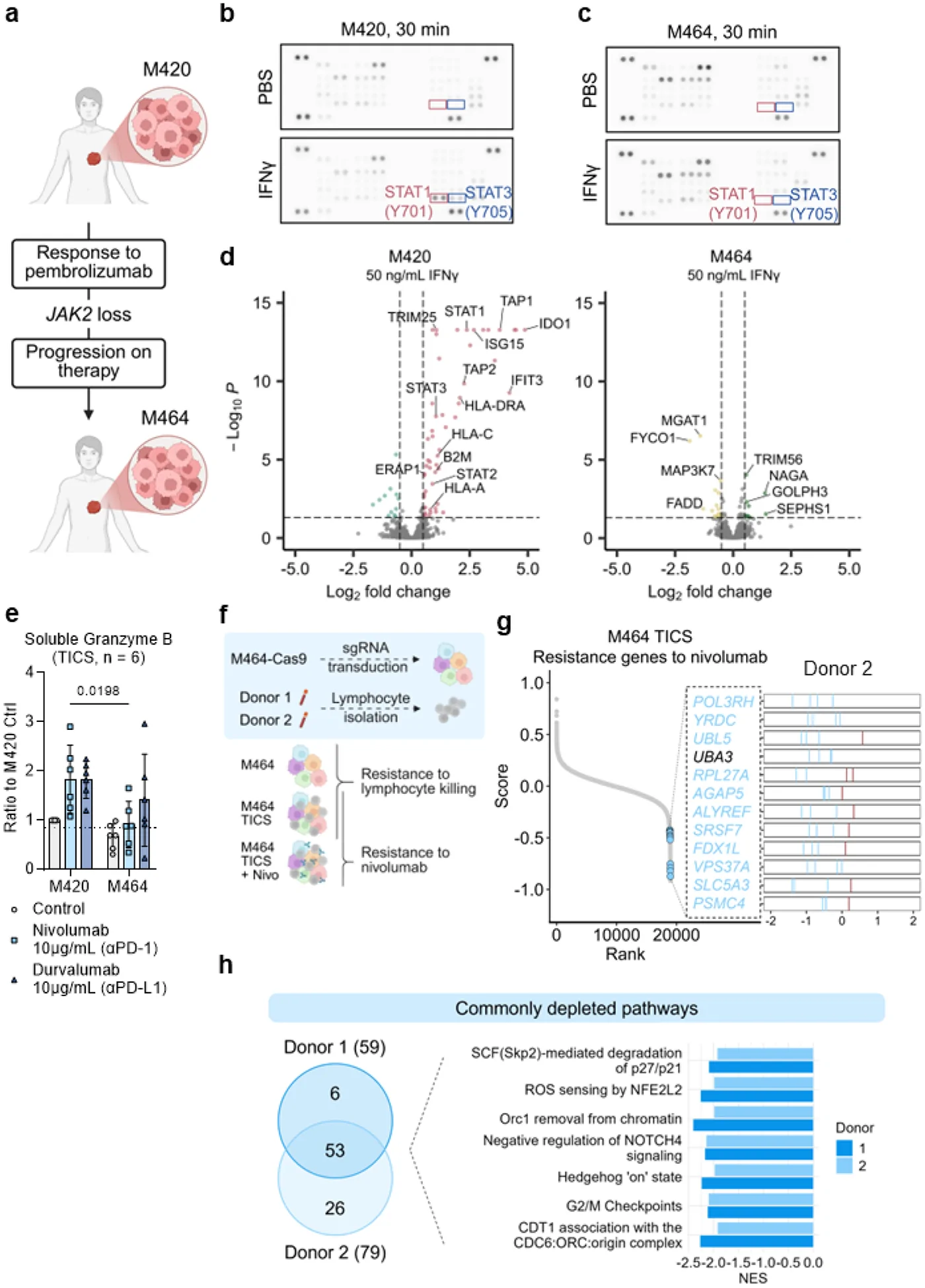
*UBA3* is a gene hit in M464 CRISPR/Cas9 screen. a) Schematic representation of the timeline at which M420 and M464 cell lines were established from tumor biopsies of a melanoma patient. b) Phospho-kinase array of M420 and c) M464 treated with 50 ng/mL of IFNγ or PBS for 30 minutes. d) Differential protein expression after 24 hours of 50 ng/mL IFNγ treatment in M420 or M464 cell lines, assessed by mass spectrometry. e) Primary human lymphocytes are co-cultured with M420 or M464 cells in the presence of nivolumab (10 μg/mL), durvalumab (10 μg/mL) or PBS. Soluble Granzyme B is quantified via ELISA in the supernatants after 6 days. Each symbol represents an individual lymphocyte donor. Mean ± SD, two-way ANOVA. f) Schematic representation of the set-up for whole-genome CRISPR screens using M464 cells in TICS. g) Gene rankplot shows depleted genes in M464 cells co-cultured with lymphocytes (donor 2) +/− nivolumab (10 μg/mL). Highlighted genes are commonly depleted in both screens. Guide RNA depletion scores for the individual guides (4 gRNAs/gene) are shown on the right panel. h) Depleted genes from each screen are selected using a cut-off at mean minus 3 standard deviations from each screen. Pathway enrichment analysis is performed using FluteRRA in reference to the Reactome database. The venn diagram demonstrates commonly depleted pathways in M464 cells from both screens.

In order to dissect cancer-intrinsic functional vulnerabilities that are specific to acquired resistance to PD1 blockade therapy, we employed a melanoma cell line pair, which was established from the same metastatic lesion in the lung, prior to pembrolizumab treatment (M420) and at disease progression after an initial response (M464) (Fig. 1a) [4]. Compared to the treatment naive M420 cell line, M464 bears a naturally-occurring new and homozygous loss-of-function mutation in the *JAK2* gene (Fig. S1a), with the resultant loss of the ability to phosphorylate STAT1 upon IFNγ stimulation (Fig. S1b). To elucidate the intracellular signaling components controlled by JAK2 in response to IFNγ, we detected 37 phosphorylation sites on a range of kinases using a protein array. In addition to STAT1, STAT3 was phosphorylated in M420 within 30 minutes of stimulation (Fig. 1b), which was not observed in M464 cells (Fig 1c, S1c).

A selected set of interferon-related mRNAs had previously been measured upon IFNγ stimulation in the M420/M464 cell line pair [4]. Therefore, we sought to comprehensively compare the translational landscape of this cell line pair at the baseline or after 24 hours of IFNγ treatment using label-free mass spectrometry (Supplementary Data 1). On average, around 3000 peptides were detected in each sample (Fig S1d). M420 demonstrated a clear change in protein expression upon IFNγ stimulation (Fig S1e), with significantly enhanced expression of IFN-response proteins (STAT1/2/3, IDO1, ISG15, IFIT3), as well as proteins in the antigen processing and presentation pathway (B2M, TAP1/2, ERAP1, HLA-A, -C, -DRA, Fig 1d). In contrast, the translational landscape remained largely unchanged in M464 cells due to the lack of JAK2 signaling (Fig. 1d, S1e). Further validation using flow cytometry showed that, although IFNγ receptor was present on the M464 cell surface and became down-regulated upon IFNγ binding, it failed to transduce the signal and up-regulate PD-L1 or HLA-ABC on the cell membrane (Fig. S1g). Of note, M420 cells presented a distinct proteome at baseline, as compared to M464 (Fig. S1f), with around 15% of the total proteins differentially expressed. We hypothesized that this difference was a result of immune-editing driven by immunotherapy, and thus performed pathway enrichment analyses on the differentially expressed proteins (M420, n=337, and M464, n=209). Our analysis showed that the treatment naïve M420 cells exhibited enriched metabolic and oxidation pathways, while M464 cells demonstrated increased cell cycle and DNA repair proteins in cell culture (Fig. S1h).

Our human Tumor-Immune co-Culture System (TICS) is specifically designed to investigate cancer-driven immune activation primed by clinically approved PD1/L1 blocking antibodies. After multiple steps of optimizations, TICS has been employed to uncover resistant genes or pathways to immunotherapy in IFN-competent human cancer cells [21, 22]. Here, we asked whether this workflow could be utilized to uncover cancer-intrinsic vulnerabilities to revert acquired immunotherapy resistance in M464 cells. Indeed, M420 elicited substantially higher granzyme B release from primary lymphocytes in the presence of either nivolumab (αPD1) or durvalumab (αPD-L1) (Fig. 1e), as compared to co-cultures with the M464 cells. This validated TICS as a relevant *in vitro* model for acquired ICB resistance, which we leveraged to complete two independent genome-wide genetic screens using M464 cells (Fig. 1f). Quantification of soluble factors in culture supernatants of the flasks in our screens verified the baseline immune activation in co-cultures and the lack of response to nivolumab (Fig. S2a and S2b). By comparing co-cultures treated with or without nivolumab, and using a cut-off value of mean minus 3 standard deviations, we identified depleted genes that could be targeted to overcome acquired resistance due to the loss of JAK/STAT signaling (Supplementary Data 2 and 3). By overlapping results from two independent screens, we obtained a total of 12 top candidate genes (*POL3RH, YRDC, UBL5, UBA3, RPL27A, AGAP5, ALYREF, SRSF7, FDX1L, VPS37A, SLC5A3, PSMC4*) (Fig. 1g, S2c). Examination of the individual screens using mainstream algorithms designed for CRISPR screens revealed that, while few genes were commonly depleted, several overlapping pathways were identified (Fig. 1h, Supplementary Data 4). Notably, *UBA3*, which is the selective E1 enzyme for the neddylation pathway, was among the top hits (Fig. 1g). Of note, neddylation pathway is known to have a major role in cancer cell proliferation and regulates several of the commonly depleted pathways, e.g. CDT1 complex and oxidative stress [23]. Because protein neddylation can be targeted to enhance response to ICB therapy in IFN-competent cells [21, 24], we propose that it could be a master regulator for both primary and acquired resistance to immunotherapy.

### Genetic deletion of *UBA3* enhances immunogenicity of *JAK2*-deficient M464 cells

**Figure 2:**
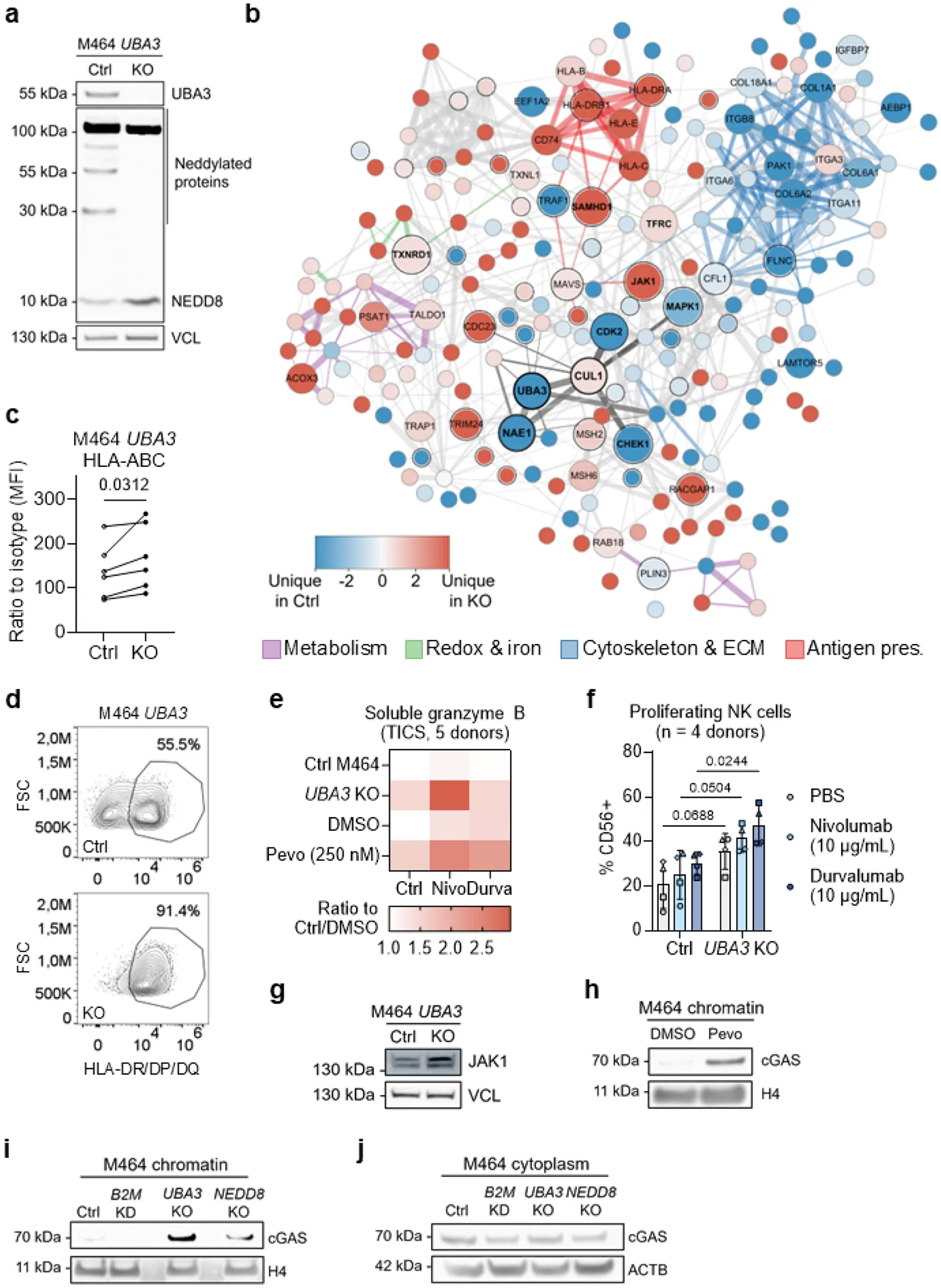
*UBA3*deletion increases antigen presentation and stabilizes chromatin-bound cGAS. a) RNP complex targeting the *UBA3* gene is transfected in M464 cells using electroporation and the expression of UBA3 and NEDD8 proteins are assessed using western blotting. b) Protein-protein interaction network comparing proteomics results from M464 control and *UBA3* KO cells. Red and blue nodes represent up-regulated or down-regulated proteins in KO cells, respectively. Nodes with black circles indicate proteins that are controlled by ubiquitin-like post-translational modifications. The width of the connecting line indicates strength of association. Proteins are grouped according to the overrepresented pathways. c) The expression of surface HLA-ABC or d) HLA-DR/DP/DQ on M464 control or *UBA3* KO cells is determined by flow cytometry. The expression levels of HLA-ABC are normalized to cells stained with an isotype control antibody. Representative flow cytometry plot for HLA-DR/DP/DQ of at least 3 biological repeats. e) Lymphocytes from 5 blood donors are co-cultured with M464 control or *UBA3* KO cells in the presence of PBS, nivolumab, or durvalumab at 10 μg/mL. On day 3, pevonedistat (250 nM) or DMSO were added. Soluble granzyme B in culture supernatants is quantified by ELISA on day 6. Mean ± SD, two-way ANOVA. f) Lymphocytes pulsed with a CellTrace Violet dye (4 donors) are co-cultured with M464 control or *UBA3*KO cells in the presence of PBS, nivolumab, or durvalumab at 10 μg/mL for 6 days and proliferation of NK cells was assessed by flow cytometry. Each symbol represents an individual lymphocyte donor. Mean ± SD, two-way ANOVA. g) JAK1 expression in M464 Control and *UBA3* KO assessed by western blot. Representative blot of at least 2 biological repeats. M464 cells are treated with h) pevonedistat treatment (250 nM) for 1 hour or i) upon *NEDD8* or*UBA3* gene deletions, and the cGAS expression is determined in the chromatin-bound fraction of cell lysate using western blotting. j) cGAS expression in the cytoplasmic fraction upon *UBA3* KO or *NEDD8* KO. A *B2M* knock-down was used to control for the effects of introduction of foreign RNA. Representative blot of at least 2 biological repeats.

To determine whether the lack of *UBA3* gene could revert acquired resistance to ICB, we deleted this gene from M464 cells using CRISPR/Cas9 knock-out (KO) technology. As expected, the loss of UBA3 enzyme prevented the conjugation of NEDD8 protein to its substrates, which led to the accumulation of free NEDD8 protein (~9 kDa) (Fig. 2a). Of note, the proliferative capacity of M464 cells were not impacted once the protein deletion was completed (Fig. S3a). Because protein neddylation is a key post-translational regulator, we examined the proteomes of control or KO cells using mass spectrometry (Supplementary Data 5). The lack of neddylation rewired the proteome in M464 cells and caused a clear separation between control and KO cells in the principal component analysis (Fig. S3b, S3c). Our proteomics analysis confirmed the absence of UBA3 protein in KO cells and identified strongly up-regulated the antigen presentation machinery (HLA-B, -C, -DRA, -DRB1, -E, and CD74) (Fig. 2b). On the contrary, cell cycle and DNA repair mechanisms (CDK2, MAPK1, and CHEK1), extracellular membrane proteins, mainly integrins and collagens, as well as cytoskeleton proteins (FLNC and PAK1) were reduced upon UBA3 deletion (Fig. 2b).

To validate our findings, we performed additional flow cytometry and western blotting experiments on the control and KO cells. Indeed, HLA-ABC was significantly upregulated on KO cells (Fig. 2c) and more than 90% of M464 cells became HLA-DR/DP/DQ positive upon *UBA3* deletion (Fig. 2d). Next, we asked whether these cellular changes could lead to stronger immune activation in TICS. As shown in Fig. 2e, genetic deletion of *UBA3* in M464 cells, or adding the neddylation inhibitor pevonedistat in the co-culture, enhanced the release of granzyme B in response to ICB drugs. When analyzing the activation of immune cell subsets by assessing their proliferation (Fig. S3d), we observed that NK cells (Fig. 2f), rather than T cells (Fig. S3e, S3f), became more activated against KO cells.

To uncover potential cancer-intrinsic mechanisms underlying the enhanced immune activation, we performed pathway enrichment analysis of the proteomics results and showed that KO cells reduced the cytoskeleton organization but rewired cellular metabolism and response to the adaptive immunity (Fig. S3g, S3h, Supplementary Data 6). Moreover, interferon pathways were among the significantly enriched pathways in KO cells (Fig. S3h), which coincided with the elevated expression of MAVS and JAK1 in KO cells (Fig. 2b, 2g). However, these changes were independent of extrinsic IFNγ signaling, as treatment of KO cells with IFNγ did not elicit the expression of IFN-responsive proteins (Fig. S3i).

Because post-translational mechanisms were recently shown to regulate cell intrinsic IFN signaling through nucleus cGAS [25], we hypothesized that inhibition of neddylation could bypass the JAK/STAT signaling by recruiting the dsRNA and/or dsDNA sensory machinery. In line with this hypothesis, pevonedistat treatment (Fig. 2h), or genetic ablation of the neddylation pathway by targeting *UBA3* or *NEDD8* (Fig. S3j), stabilized the chromatin-bound cGAS in M464 cells (Fig 2i), without altering the expression of cGAS in the cytoplasm or the soluble nuclear fraction (Fig 2j. S3k).

### Blocking neddylation *in vivo* restores the response to PD1 blockade in *B2m* and/or *Jak1/2*-deficient cancer cells

**Figure 3:**
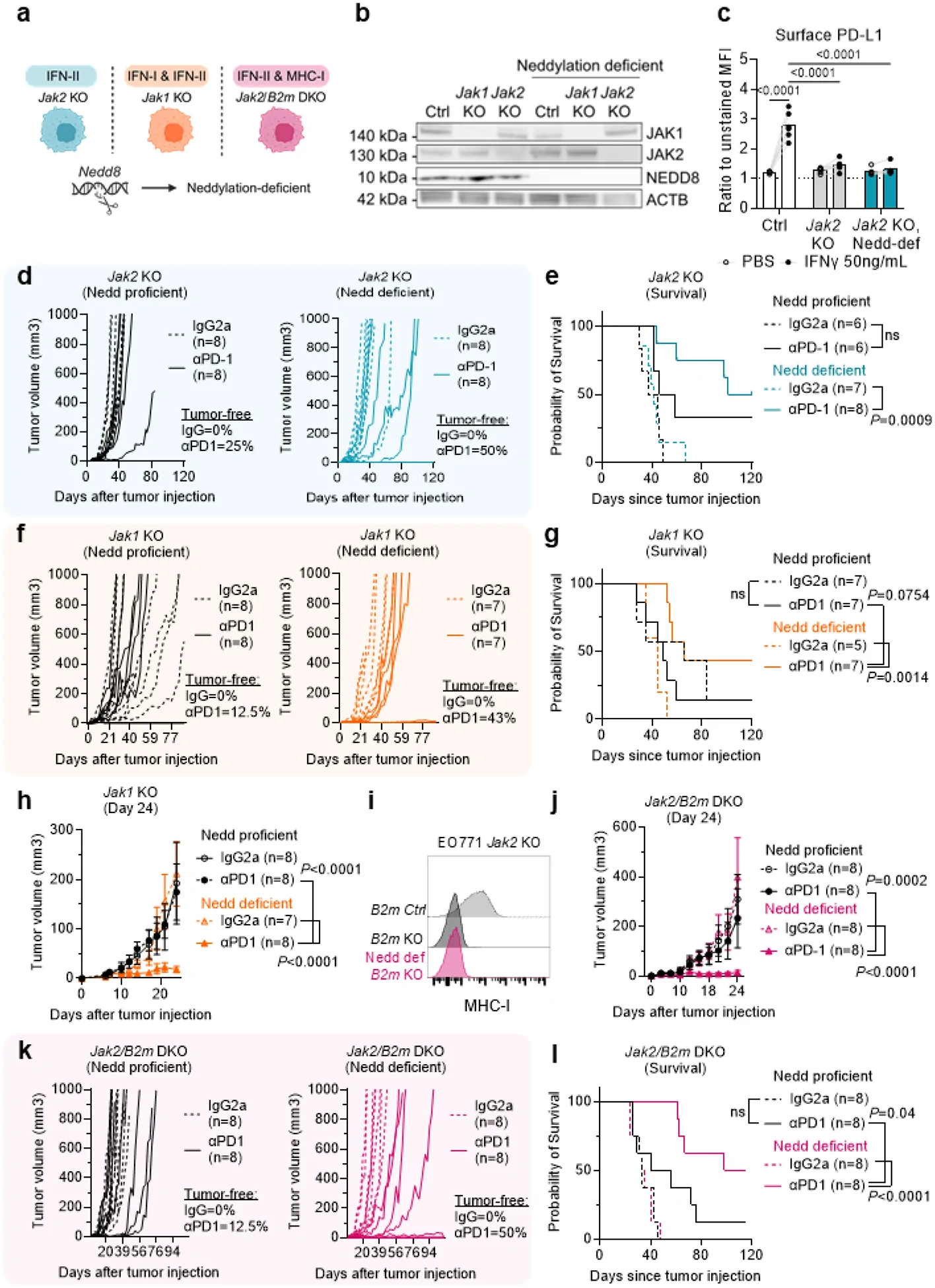
Genetic deletion of neddylation in cancer cells reverts acquired resistance to immunotherapy. a) Schematic summary of isogenic cancer cell line pairs with different combinations of genetic deletions in murine EO771 cells. All cell lines are generated without single cell cloning. b) Confirmation of the lack of protein expression for JAK1, JAK2, and NEDD8 in KO cells using western blotting. c) PD-L1 expression is quantified using flow cytometry after *in vitro*stimulation with 50 ng/mL recombinant mouse IFNγ for 24 hours. Bars show the average ratio to unstained control cells treated with PBS. Two-way ANOVA was used for statistical analysis. d) Six to ten weeks old female C57BL/NTac mice were injected subcutaneously with 600,000 EO771 cells. Growth of neddylation-proficient (black) or -deficient (blue), *Jak2* KO tumors treated with PD1 blockade or an isotype control IgG is shown. Each line represents an individual mouse. e) Survival probability is calculated with log-rank (Mantel-Cox) test for tumor-bearing mice treated with PD1 blockade or an isotype control. f) Growth of neddylation-proficient (black) or - deficient (orange), *Jak1* KO tumors treated with PD1 blockade or an isotype control IgG is shown. Each line represents an individual mouse. g) Survival probability was calculated with log-rank (Mantel-Cox) test for mice bearing tumors with the *Jak1* KO background. h) Tumor growth of tumors with *Jak1*deficiency up to day 24. Lines and dots represent the average tumor size for each group ± SEM. 2-way ANOVA with Bonferroni correction was used to determine p values at each timepoint. i) *Jak2* and/or neddylation-deficient EO771 cells are transfected with an RNP complex that targets the mouse *B2m*gene. The KO cells are selected by FACS. Flow cytometry analysis of H2-KD protein expression in the cell lines. Representative flow cytometry plot of at least two biological repeats is shown. j) Growth of neddylation-proficient (black) or -deficient (pink), *Jak2/B2m* DKO tumors treated with PD1 blockade or an isotype control IgG is shown up to day 24. Lines and dots represent the average tumor size for each group ± SEM. 2-way ANOVA with Bonferroni correction is used to determine p values at each timepoint. k) Growth of neddylation-proficient (black) or -deficient (pink), *Jak2/B2m* DKO tumors treated with PD1 blockade or an isotype control IgG is shown. Each line represents an individual mouse. l) Survival probability was calculated with log-rank (Mantel-Cox) test for mice bearing tumors with *Jak2/B2m* DKO background. All *in vivo* studies are repeated in at least two biological replicates.

To test whether neddylation can be a vulnerability against genetic defects on the antigen presentation and IFN pathway in mice, we first optimized the treatment dose and schedule in the EO771 syngeneic murine tumor model to achieve a high cure rate (>60%) upon PD1 blockade (Fig. S4a, S4b, S4c). Deletion of the *Jak2* gene in cancer cells conferred resistance to the therapy in this model, due to the lack of intra-tumoral IFNγ signaling amplification (Fig. S4d, S4e), and reduced the long-term survival rates (Fig. S4f, S4g). Moreover, we created a panel of cancer cell lines that were deficient on the JAK/STAT pathway (*Jak1* or *Jak2* KO) or with the additional loss of the MHC-I antigen presentation (*Jak2/B2m* double KO, DKO) (Fig. 3a). The *Nedd8* gene, which is required for protein neddylation [23], was genetically ablated in each KO background using CRISPR/Cas9 (Fig. 3b). To preserve the cell line heterogeneity, gene deletions were accomplished without single cell cloning. Similar to human cancer cells, the JAK/STAT signaling was required for the IFNγ-driven up-regulation of surface PD-L1 on EO771 cells, which was not influenced by neddylation deficiency (Fig. 3c).

In line with our hypothesis, genetic deletion of neddylation in murine cancer cells did not impact tumor growth, but it sensitized *Jak2*-deficient tumors to PD1 blockade (Fig. 3d), and significantly prolonged the survival in tumor-bearing mice (Fig. 3e). Different to *Jak2*-deficient tumors, where an early tumor growth delay was recorded upon PD1 blockade (Fig. S4h), *Jak1* deficiency in cancer cells caused a complete resistance to PD1 blockade, which was significantly reverted in neddylation-deficient tumors (Fig. 3h). This was translated to superior responses and survival benefits to PD1 blockade in these mice (Fig. 3f, 3g).

Because *Jak1/2*-deficient cancer cells can still be recognized by cytotoxic T cells, we asked whether the ablation of neddylation is sufficient to overcome severe immune resistance when both JAK/STAT signaling and MHC-I antigen presentation are disabled in the *Jak2/B2m* DKO cells (Fig. 3i). Strikingly, neddylation deficiency elicited potent anti-tumor immunity against *Jak2/B2m* DKO tumors upon PD1 blockade (Fig. 3j), and half of the mice in the group were tumor-free (Fig. 3k). This led to a significant survival benefit in this group, as compared to neddylation proficient tumors treated with PD1 blockade (Fig. 3l)

### Innate immune cells, but not T cells, are more attracted to neddylation-deficient tumors

**Figure 4:**
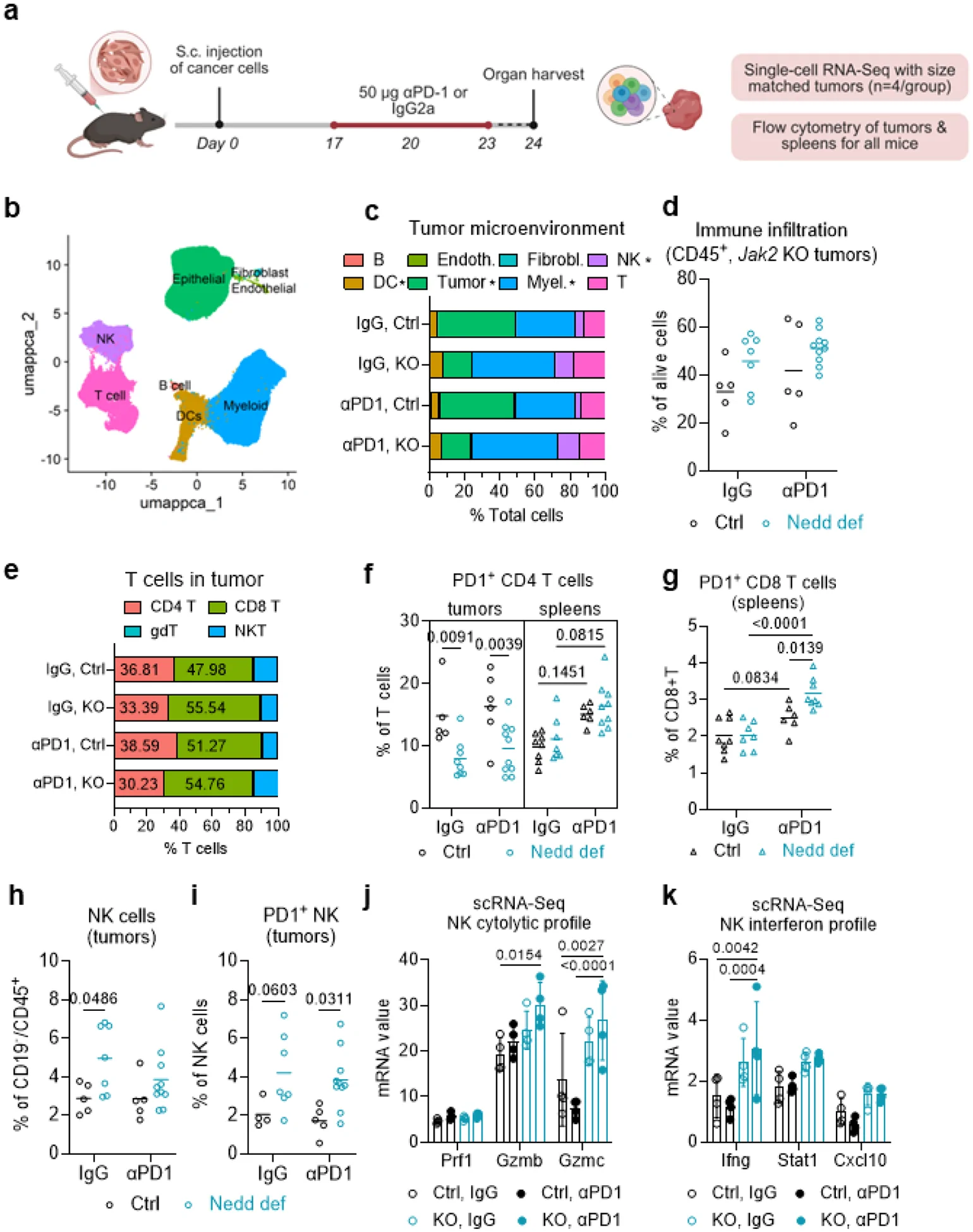
Single-cell analysis of *Jak2* KO tumors with neddylation deficiency. a) Schematic representation of the setup for the experimental procedure. Treatment of neddylation-proficient or -deficient on the *Jak2* KO background with αPD1 or isotype control is started when average tumor volume reached >50mm^3^ (day 17). Tumors and spleens are harvested on day 24 post-injection, one day after the last dose of αPD1 or the isotype control. b) UMAP of cell clustering in *Jak2* KO tumors according to data from scRNA-Seq. c) Average percentage of each cell type in relation to total amount of cells from scRNA-Seq in each group. Stars indicate statistically significant differences in the cellular portions between neddylation proficient and deficient tumors, evaluated by Wilcoxon rank sum test. d) Flow cytometry analysis of tumor-infiltrating CD45+ immune cells in the live cell population. Each dot represents an individual tumor. Statistical differences are assessed with 2-way ANOVA and Bonferroni correction. e) Average percentage of tumor-infiltrating T cell subsets in different groups, according to scRNA-Seq. f) Percentages of PD1+ cells in intra-tumoral or splenic CD4+ T cells, g) PD1+ cells in splenic CD8+ T cells, h) Intra-tumoral NK cells in CD45+ cells, and i) PD1+ cells in intra-tumoral NK cells are assessed by flow cytometry, and statistical significance was calculated using 2-way ANOVA and Bonferroni correction. Each dot represents an individual tumor (point) or spleen (triangle). j) mRNA expression of cytolytic genes and k) interferon genes in tumor-infiltrating NK cells, assessed by scRNA-Seq. Differences are tested using 2-way ANOVA and Bonferroni correction. Each dot represents an individual tumor.

To discern immunological changes that drive the superior response against acquired resistance, we combined single-cell RNA sequencing (scRNA-Seq) and flow cytometric analyses on *Jak2*-deficient control or *Jak2*/neddylation-deficient tumors. In order to obtain sufficient tumor materials, treatments were initiated on larger tumors and cells were isolated one day after the third dose of anti-PD1 antibody or the IgG control (Fig. 4a and S4i). Immune and epithelial cells were the dominant cell types in *Jak2*-deficient tumors according to scRNA-Seq (Fig. 4b). To determine the abundance of cancer cells within the epithelial cell population, we inferred the copy number variation (CNV) and observed that 99% of the cells were malignant (Fig. S5a). Tumors lacking functional protein neddylation demonstrated lower cancer cell load regardless of the treatment group (green in Fig. 4c), compared to the control counterparts. This could be a result from the enhanced immune cell infiltration in these tumors (Fig. 4d). Among the immune cell subsets, intra-tumoral innate immune cells were more abundant in neddylation-deficient tumors, specifically dendritic cells (Fig. 4c, KO vs Ctrl, IgG=7.5 % vs 4.31%; KO vs Ctrl, αPD1=7.18% vs 4.25%; Wilcoxon test, p = 0.057 for IgG, p = 0.029 for αPD1), NK cells (KO vs Ctrl, IgG=10.43 % vs 4.3%; KO vs Ctrl, αPD1=12 % vs 3.75 %; Wilcoxon test, p = 0.029 for IgG, p = 0.029 for αPD1) and myeloid cells (KO vs Ctrl, IgG=47.43 % vs 33.75%; KO vs Ctrl, αPD1=48.95 % vs 33.92 %; Wilcoxon test, p = 0.029 for IgG, p = 0.057 for αPD1).

In contrast, neddylation-deficient tumors did not result in changes in the percentages of infiltration of T cells (Fig. S5b, S5c), nor the balance among CD8+ T, CD4+ T, gamma-delta (γδ) T, or NKT lymphocyte subsets (Fig. 4e, S5d, S5e, S5f). Sub-clustering of CD8+ T cell subsets did not reveal significant changes in naïve, exhausted, effector or memory phenotypes between control and knock-out or the IgG and αPD1 treated tumors (Fig. S5g, S5h, S5i). Intra-tumoral CD4+ T cells were mostly regulatory, and similar to the CD8+ T cell compartment, the populations remained unchanged upon treatment and between the control and KO groups (Fig. S5j, S5k, S5l). However, PD1 expression on CD4+ T cells was increased in neddylation-proficient tumors, but remained unchanged upon PD1 blockade (Fig. 4f). PD1 blockade led to increased expression of PD1 on splenic CD4+ T cells and this effect was significant in mice bearing KO tumors (Fig. 4f, S6a, S6b). Similarly, PD1 expression on intra-tumoral CD8+ T cells was comparable among groups (Fig. S6c), but was elevated in splenic CD8+ T cells from mice (Fig. 4g). Notably, the increased infiltration of NK cells as a result of neddylation deficiency was verified by flow cytometry (Fig. 4h), and these cells elevated the expression of PD1 on the cell surface (Fig. 4i). Moreover, intra-tumoral NK cells in neddylation-deficient tumors expressed higher levels of cytolytic genes (*Gzmb, Gzmc*) (Fig, 4j) and interferon response genes (*Ifng, Stat1, Cxcl10*) (Fig. 4k, S6d), as compared to control tumors (Fig. S6d, S6e).

### Antigen presenting cells show pro-inflammatory phenotypes in neddylation-deficient tumors

**Figure 5:**
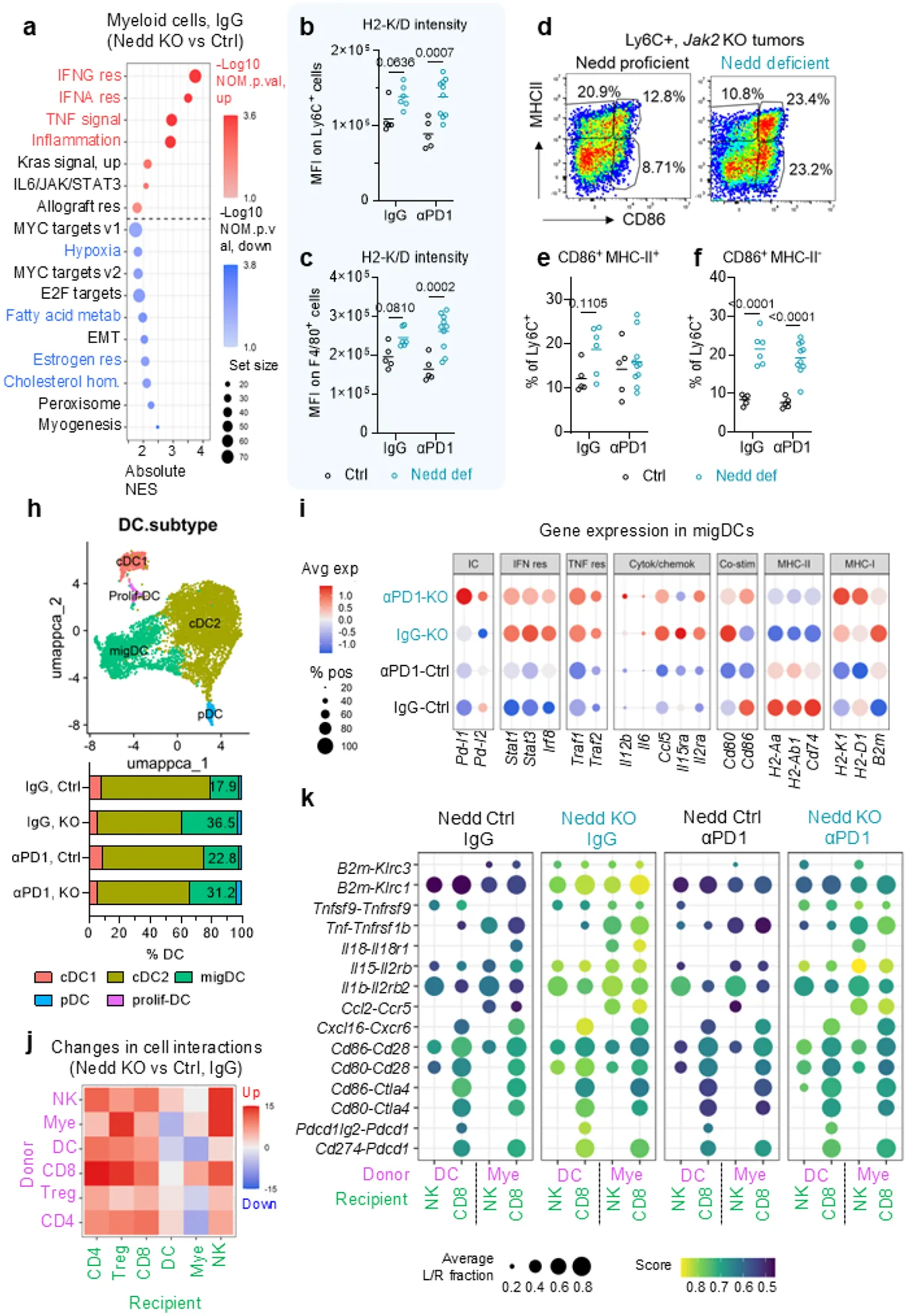
Pro-inflammatory myeloid cells and migratory dendritic cells are enriched in neddylation-deficient tumors. a) GSEA pre-ranked pathway analysis of differentially expressed genes is performed in myeloid cells from neddylation-deficient or proficient tumors treated with the isotype control. b) H2-KD intensity on Ly6C+ or c) F4/80+ myeloid cells, assessed by flow cytometry. Each dot represents an individual tumor. Differences are compared using 2-way ANOVA and Bonferroni correction. d) Expression of MHC-II and CD86 is assessed in the Ly6C+ intra-tumoral myeloid cells by flow cytometry. Representative flow cytometry plot or the percentages of e) CD86+MHC-II+ or f) CD86+MHC-IIneg subsets are compared among groups. Each dot represents an individual tumor. Statistical significance is calculated using 2-way ANOVA and Bonferroni correction. h) Cell clustering of intra-tumoral dendritic cell subtypes, using cells from all tumors analyzed in scRNA-Seq. The stacked bar plot shows differences per-group, with each bar representing the average of each subset in each group. i) Relative mRNA expression of cytokines, chemokines, TNF, IFN, antigen presentation and checkpoint ligands in migDC from different groups in scRNA-Seq. Values are z-transformed, and a higher expression is represented in red. The size of the dots represents the percentage of cells that express that gene. j) Heatmap represents the number of up-regulated interactions subtracted by the number of down-regulated interactions in each cell pair in *Jak2* KO tumors treated with the isotype control. Down/up-regulation of interactions are defined as described in methods. Y axis shows the donor cell type and the X axis shows the recipient cell type. Red boxes represent enhanced overall cell-cell interactions while blue boxes represent reduced interactions. k) Predicted ligand-receptor interactions between donor DC/myeloid cells and recipient CD8+ T or NK cells, calculated using the NicheNet algorithm. Color represents strength of the interaction, and size of the dot represents the combined percentage of donor and recipient cells expressing the ligand and receptor, respectively.

Because myeloid cells were abundant and heterogenous in the *Jak2*-deficient mouse tumors, we performed pathway analysis by comparing myeloid cells in neddylation-deficient and -proficient tumors. We observed that myeloid cells were more pro-inflammatory in neddylation-deficient tumors, with IFN and TNF response pathways among the top enriched pathways (Fig. 5a). In accordance, these myeloid cells down-regulated cholesterol, fatty acid, and estrogen signaling, as compared to cells from neddylation-proficient tumors, which could suggest reduced immunosuppressive capacity [26]. Flow cytometric analysis (Fig. S7a) showed that, while percentages of myeloid cell subsets were comparable among groups (Fig. S7b, S7c, S7d), both Ly6C+ monocytes (Fig. 5b) and F4/80+ macrophages (Fig. 5c) expressed MHC-I molecule at a higher level in neddylation-deficient tumors, which was confirmed in scRNA-seq (Fig. S7e). Moreover, the co-stimulatory potential of myeloid cells was enhanced, indicated by the elevated expression of CD86 (Fig. 5d, 5e, 5f, S7f). Of note, myeloid cell subsets in the spleens remain largely unchanged (Fig. S7g, S7h, S7i, S7j, S7k), with the exception that splenic monocytes (Fig. S7i) and macrophages (Fig. S7j) were slightly reduced in untreated mice bearing neddylation-deficient tumors.

Among the tumor-infiltrating DC subsets (Fig. S8a), migratory DCs (migDC) and cDC2 were elevated in neddylation-deficient tumors (Fig. S8b), although only migDC showed a significant enrichment within the DC population (Fig. 5h, S8b). Pseudo-time analyses revealed that migDC were most likely differentiated from cDC2 (Fig. S8c). MigDC showed a high expression of *Ccr7* and *Fscn1* (Fig. S8d, S8e), as well as expressed *Cd274* at a higher level, as compared to other DC subsets (Fig. S8f), confirming its migration and immune regulatory potential. When comparing differentially expressed genes in migDC, we observed a heightened activation status in neddylation-deficient tumors, with higher levels of IFN and TNF responses, as well as cytokine and chemokine genes (Fig. 5i, S8g). Similar to myeloid cells, a shift in antigen presentation was observed, where migDC from neddylation-deficient tumors demonstrated increased expression of MHC-I genes but lower levels of MHC-II genes.

Because frequencies of tumor-infiltrating T cells were not substantially changed among groups, we hypothesized that the cross-talk among the innate and adaptive immune cell types could be crucial for the superior anti-tumor immune response in neddylation-deficient tumors. We employed the multi-Nichnet algorithm to computationally stipulate cell-cell interactions based on the scRNA-seq dataset. This analysis revealed enhanced interactions among immune cell subsets (Fig. 5j), except signals received by DC or myeloid cells. In particular, DC and myeloid cells demonstrated more frequent interactions with the adaptive immunity through the MHC-I antigen presentation, CD80/86 co-stimulation and IL2/15 cytokine signaling in neddylation-deficient tumors (Fig. 5k). In the same tumors, interactions between PD1-PDL1/2 (*Pdcd1-Cd273/274*) and MHC-NKG2A/E (*B2m-Klrc1/3*) intensified between DC/myeloid cells and CD8+ T cells and/or NK cells (Fig. 5k). Given that *Pdcd1* mRNA was mostly expressed by T cells while *Cd273/274* mRNAs were enriched in migratory DC (Fig. S7a) and myeloid cells (Fig. S8h), our data suggested that PD1/L1 blockade could alleviate the immune regulatory interactions between the innate and the adaptive immune cells upon neddylation deletion in cancer cells.

### Cancer-intrinsic cGAS orchestrates the immune regulatory properties of innate immune cells.

**Figure 6:**
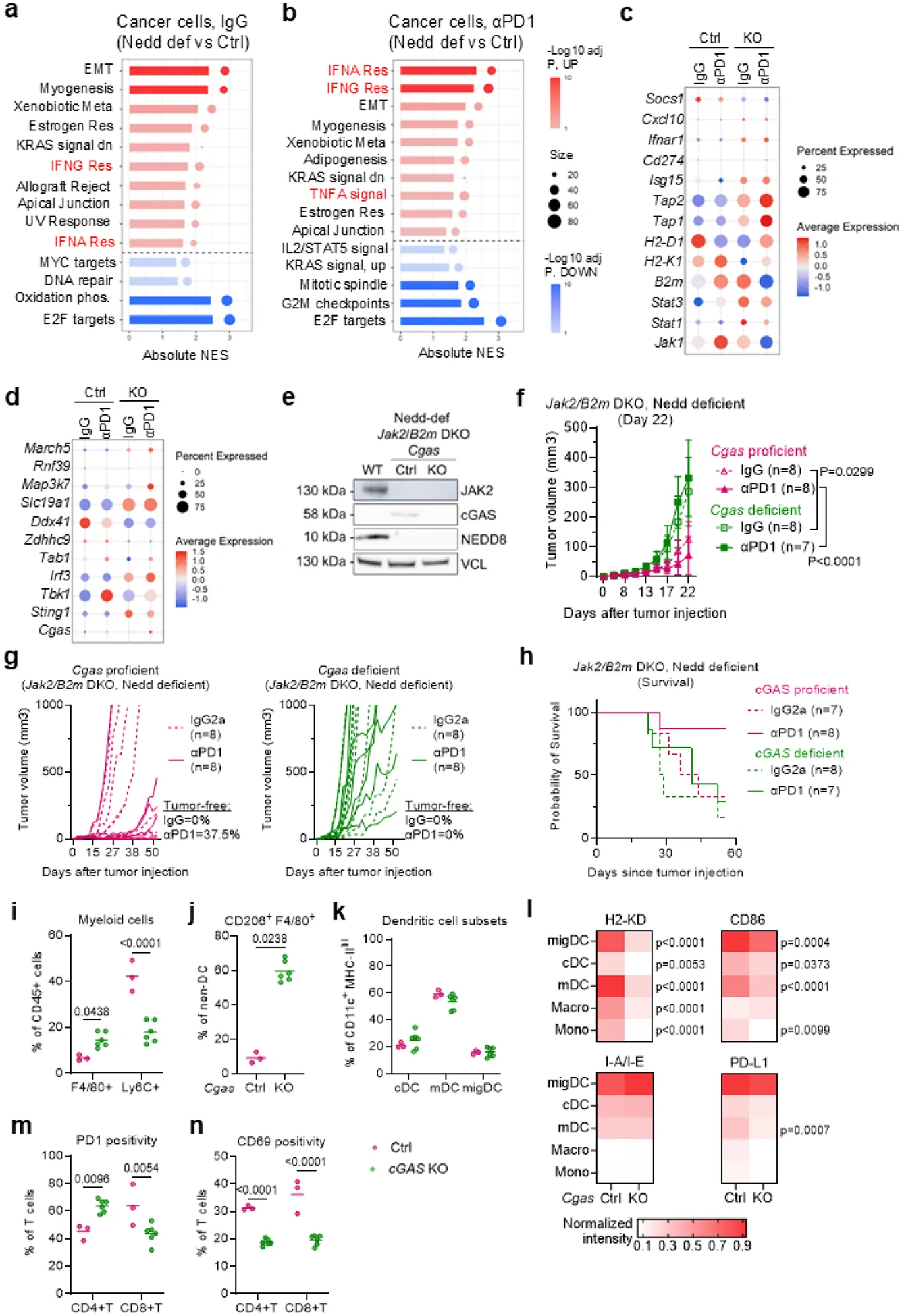
Neddylation deficiency requires cGAS to overcome resistance to PD1 blockade. GSEA pre-ranked pathway analysis of differentially expressed genes is performed in cancer cells from neddylation-deficient and -proficient in *Jak2* KO cancer cells in tumors treated with a) isotype control and b) αPD1. Pathways in red or blue represents enriched or depleted pathways in neddylation-deficient tumors, respectively. Relative expression of genes in c) the antigen presentation and interferon response pathway, or d) the cGAS/STING pathway, grouped by neddylation status and treatment groups. Dot color represents relative expression, with red being high and blue being low. Size of the dot represents the percentage of cells expressing that mRNA. e) cGAS protein expression in EO771 cells, evaluated by western blotting. Representative blot for at least two biological repeats. Control (pink) or *Cgas*-deficient (green) cancer cells generated on the *Jak2/B2m* DKO and neddylation-deficient EO771 cells are injected in C57BL/NTac mice and treated with PD1 blockade or an isotype control antibody. f) Tumor growth is shown up to day 24. Lines and dots represent the average tumor size for each group ± SEM. 2-way ANOVA with Bonferroni correction is used to determine p values. g) Growth of individual tumors until the study endpoint is shown. Each line represents an individual mouse. h) Survival probability is calculated with log-rank (Mantel-Cox) test for tumor-bearing mice in different groups. Immune cells from large mouse tumors (>1000mm^3^) at the study endpoint are analyzed using flow cytometry and i) percentages of F4/80+ or Ly6C+ myeloid cells in CD45+ cells, j) CD206+ macrophages, k) subsets of dendritic cells are shown in control or *Cgas* KO tumors. Each dot represents an individual tumor and statistical significance is calculated using 2-way ANOVA and Bonferroni correction. l) Mean fluorescence intensity of H2-KD, I-A/I-E, CD86 and PD-L1 in different DC subsets (migratory DC, classical DC, and myeloid DC), macrophages and monocytes are shown. MFI values are normalized to a [0,1] distribution, with red representing the highest expression and white the lowest MFI value. Percentages of m) PD1-expressing or n) CD69-expressing cells in intra-tumoral CD4+ or CD8+ T cells in control or *Cgas* KO tumors are shown. Each dot represents an individual tumor and statistical significance is calculated using 2-way ANOVA and Bonferroni correction.

After establishing that neddylation-deficient tumors rewired the tumor microenvironment towards a pro-inflammatory and innate-immune rich phenotype, we then assessed the underlying mechanisms in cancer cells from mouse tumors. Neddylation-deficient cancer cells exhibited enriched epithelial-to-mesenchymal transition (EMT) and estrogen response gene signatures, but reduced E2F and oxidative phosphorylation pathways, as compared to the neddylation-proficient tumors (Fig. 6a). Similar to M464 cells, deletion of neddylation enhanced IFNG and IFNA responsive genes in cancer cells (Fig. 6a). Strikingly, these pathways, as well as the TNFA signaling, became strongly enriched in neddylation-deficient cancer cells when mice were treated with PD1 blockade (Fig. 6b, S9a, S9b). For example, genes related to antigen processing (*Tap1/2, B2m*), and JAK/STAT (*Stat1/3, Isg15, Ifngr1, Cxcl10*) were increased in neddylation-deficient cancer cells (Fig. 6c, S9c). Because the JAK/STAT signaling is lost in mouse cancer cells, this could be driven by the cGAS/STING responsive genes (*Sting1, Irf3, Slc19a1, Marchf5*) (Fig 6d, Fig S9d).

To provide mechanistic insights into the role of cGAS upon neddylation deficiency, we employed the *Jak2/B2m* DKO mouse tumor model, which demonstrated a robust response to PD1 blockade in mice (Fig. 3j), and deleted the *Cgas* gene in cancer cells. In accordance to results obtained in tumor-bearing mice (Fig. 6d), cGAS protein expression was elevated in cultured neddylation-deficient cells, which was abrogated by CRISPR-based gene silencing (Fig. 6e). In mice, *Cgas* KO tumors demonstrated a more aggressive growth behavior and lost the ability to respond to PD1 blockade (Fig. 6f, 6g), which compromised the survival benefits provided by neddylation deficiency (Fig. 6h).

To test whether cancer-intrinsic cGAS is responsible for the recruitment of pro-inflammatory antigen presenting cells in large tumors, we harvested tumors when they reached the experimental endpoint (>1000mm^3^) and analyzed the intra-tumoral immune cells using flow cytometry (Fig. S10a). *Cgas* gene deletion in cancer cells caused a significant increase in macrophages (Fig. 6i, S10b), which exhibited predominantly a CD206+MHC-I^low^ anti-inflammatory phenotype (Fig. 6j, 6l). Percentages of DC subsets were comparable between *Cgas*-proficient and -deficient tumors (Fig. 6k), but showed impaired expression of MHC-I and CD86 in *Cgas*-deficient tumors (Fig. 6l). Moreover, the phenotypic changes in innate immune cell subsets coincided with differential expression of receptors on tumor-infiltrating T cell subsets. PD1-expressing cells were significantly more prevalent in CD4+T cells from *Cgas*-deficient tumors, but reduced in CD8+ T cells (Fig. 6m). Moreover, both CD4+ and CD8+ T cell subsets showed significantly reduced expression of the activation marker CD69, if *Cgas* was not present in cancer cells (Fig. 6n).

### Protein neddylation in cancer cells is associated with migDC frequencies in patients with melanoma

**Figure 7:**
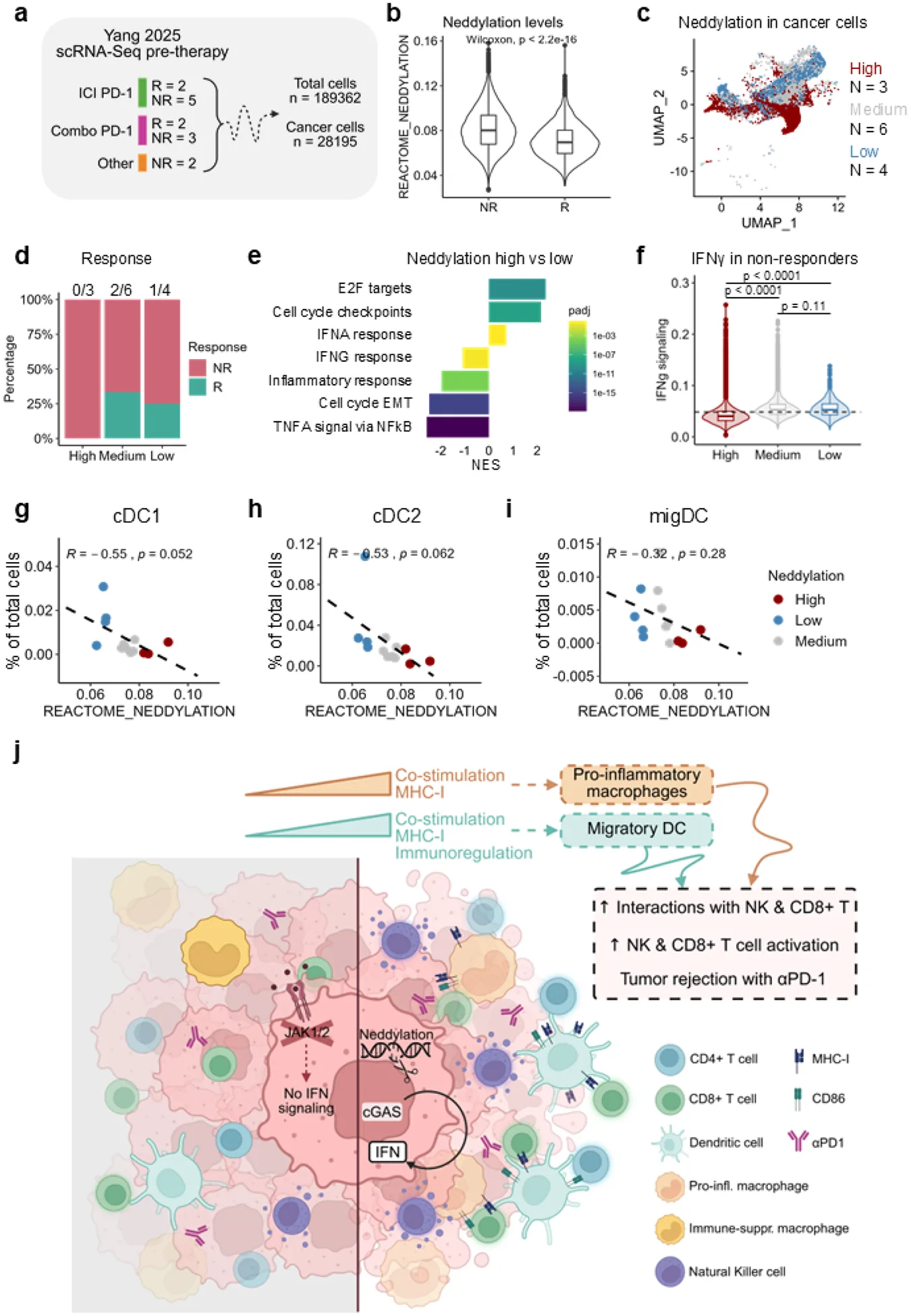
Dendritic cell infiltration is associated with cancer-intrinsic neddylation scores in human melanoma samples. a) Schematic representation of scRNA-Seq dataset and sample features. Only cancer cells were used for subsequent analysis. b) Neddylation scores in cancer cells are calculated with AUCell and grouped based on response to immunotherapy (NR: non-responders; R: responders). Statistical difference was calculated with the non-parametric Wilcoxon test. c) Cancer cells from all patient samples are pooled and grouped according to the neddylation score. One patient (ICI PD1, R) is removed from analysis as it only had 8 cancer cells sequenced. Each dot represents a single cancer cell. d) Stacked bar plot showing the percentage of neddylation high, medium and low patients and their associated response. e) Pathway enrichment analysis of differentially expressed genes when comparing cancer cells from neddylation high (n=3) and low (n=4) patients. X axis shows the normalized enrichment score (NES) and positive values represent more enriched pathways in neddylation high patients. Color of the bar represents the adjusted p value with False Discovery Rate. f) IFNg signaling pathway enrichment is calculated at the single cell level in all cancer cells of non-responding tumors using AUCell. Cells are grouped according to their neddylation scores. Differences are calculated using Wilcoxon rank sum test. The percentages of g) cDC1, h) cDC2, i) migDC in patient tumors are correlated with the average neddylation scores in cancer cells from the same sample. The correlation values are calculated using Pearson correlation. j) Graphical summary of the mechanisms underlying the potent immune activation network upon genetic deletion of protein neddylation in cancer cells.

To infer on the relationship between cancer-intrinsic neddylation and dendritic cells in human cancer samples, we then used a publicly available scRNA-Seq dataset from therapy-naïve patients with melanoma [27] (Fig. 7a, S10c). Firstly, we calculated the intrinsic neddylation scores in cancer cells, and demonstrated that tumor biopsies from responders to PD1 blockade, either as a monotherapy or in combination, contained cancer cells with lower neddylation values (Wilcoxon test, p < 2.2 × 10^−16^) (Fig. 7b). We then stratified patients based on the average levels of cancer-intrinsic neddylation scores (Fig. S10d) and separated patients into high (n=3), medium (n=6) or low (n=4) groups (Fig 7c). None of the neddylation-high patients had a favorable response in their lesions, while 1/4 of the low neddylation tumors and 2/6 of the medium neddylation tumors benefited from therapy (Fig. 7d). Despite this, this stratification did not correlate with patient survival (Fig. S10e). Next, we performed differential gene expression (Fig S10f) and pathway enrichment analysis in cancer cells between patients with high or low neddylation scores. This analysis revealed that pathways that were enriched in neddylation-proficient murine tumors (Fig. 6a, 6b), such as E2F targets and cell cycle checkpoints, were also enriched in neddylation-high patients (Fig. 7e). Moreover, EMT, inflammatory processes and TNFA signaling pathways, which were enriched in neddylation-deficient murine tumors (Fig. 6a, 6b), were elevated in patient tumors with low neddylation scores (Fig. 7e). However, interferon responses were not differentially expressed between neddylation-high and -low patients, potentially due to the JAK/STAT pathway competence in the human samples.

We further assessed whether neddylation scores could be associated with interferon signaling in human tumors by calculating the interferon gamma signaling in a per-cell manner and compared them across neddylation groups in non-responding tumors (Fig. 7f). Patients with high neddylation scores in cancer cells had a lower average of interferon signaling pathway enrichment compared the medium and low patients. Finally, we tested whether neddylation in cancer cells could be correlated with the infiltration of dendritic cells in human melanoma. Although dendritic cell subsets were at low abundance in patient tumors, they inversely correlated with the average neddylation levels in cancer cells (cDC1, R = −0.55, Fig 7g; cDC2, R = −0.53, Fig. 7h, and migDC, R = −0.32, Fig. 7i).

Altogether, our study provides a comprehensive analysis to highlight the therapeutic potential of protein neddylation in reverting resistance to immunotherapy. The loss of neddylation activates the intracellular signaling network in cancer cells and bypass the JAK/STAT signaling pathway to achieve curative effects. This mechanism relies on the cGAS/STING pathway and the activation and recruitment of pro-inflammatory innate immune cells (Fig. 7j).

## Discussion

Loss of cancer-intrinsic JAK/STAT signaling and MHC-I antigen presentation dampens response to immunotherapy and can lead to primary or acquired resistance [2, 16]. Using genome-wide functional screens, we identify cancer-intrinsic protein neddylation as a gatekeeper for primary and acquired immunotherapy resistance. Genetic targeting of the neddylation pathway rewires the intracellular immunogenic network of JAK/STAT-deficient cancer cells, and reverts acquired resistance to PD1 blockade in tumor-bearing mice. Protein neddylation is a post-translational machinery that sustains the uncontrolled proliferation of cancer cells [23]. Recent results demonstrate its potential to sensitize cancer cells to immunotherapy [21, 24, 28], but the mechanistic link between neddylation, interferon signaling, and acquired resistance to immunotherapy remains unknown.

We performed in-depth analysis at the single-cell level of human and murine tumors, and show that genetic deletion of neddylation enhances the transcription of IFN response and immune-stimulating genes in cancer cells, which potentiates an influx of antigen presenting innate immune cells in tumors. For example, pro-inflammatory CCR7+ migratory DC, cDC2, and macrophages with immune-stimulatory phenotypes are attracted to the tumor microenvironment, which could promote the spatial re-organization and recruitment and priming of cytotoxic immune cells through the release of chemokines [29, 30]. Moreover, NK cells are better activated by neddylation-deficient M464 cells in human co-cultures, and NK cells are found at increased percentages and with a heightened cytotoxic profile in neddylation-deficient murine tumors. Given that *Jak2/B2m* DKO cancer cells can be eliminated by PD1 blockade upon neddylation deletion, the direct cytotoxicity of NK cells against cancer cells, as well as their interplay with DC subsets [30, 31] should be further investigated in these models.

Despite the highly pro-inflammatory status in cancer and innate immune cells, neddylation-deficient tumors grow comparably to the control tumors at the baseline, regressing only upon PD1 blockade treatment. By computationally stipulating cell-cell interactions, we observed intensified crosstalk between the innate and adaptive immunity. In particular, antigen presenting cells more frequently engaged with T cells through antigen presentation and co-stimulatory pathways, but also expressed PD-L1 and PD-L2 at high levels. The interplay between the inhibitory receptors and ligands could dampen anti-tumor adaptive immunity and explain why PD1 blockade is necessary for tumors to be eliminated. This assumption reconciles with several earlier studies, where PD-L1 on intra-tumoral DC plays an essential regulatory role to anti-tumor immunity in mice and was responsible for the response elicited by ICB therapy [32–35]. Moreover, several recent studies proposed that high percentage of migDC was associated with favorable responses to PD1 blockade therapy in patients [27, 35–38], and our analysis further demonstrate that DC subsets are more abundant, if the patient tumors contain cancer cells with lower neddylation pathway activity. However, additional patient tumor samples, especially tumors with the loss of JAK/STAT signaling, should be included to strengthen the clinical relevance of our study on acquired immunotherapy resistance.

The cGAS/STING pathway is a cellular machinery in sensing aberrant DNAs, which fuels the activation of anti-tumor immunity through the type I IFN pathway [39]. In accordance, restoration of the cGAS/STING signaling by mRNA transfection in cancer cells increased the antigen recognition by T cells [40], and recruited highly activated DCs and myeloid cells in tumor-bearing mice [41]. When investigating mechanisms underlying the restored IFN signaling upon neddylation deficiency, we found that the chromatin-bound cGAS was stabilized in human and murine *JAK2*-deficient cancer cells. This is in line with a recent study [25], where the availability of nuclear cGAS is tightly controlled by the post-translational machinery. Pharmacological inhibition of protein neddylation by pevonedistat, or genetic deletion of the key components on the post-translational machinery, stabilized chromatin-bound nuclear cGAS, which could enhance the transcription of interferon-related genes in cultured human cancer cells. Here, our findings highlight the *in vivo* relevance of this mechanism, and demonstrate that the superior anti-tumor efficacy in neddylation-deficient tumors is mechanistically dependent on the presence of cGAS protein in cancer cells. Because chromatin-bound cGAS was shown to enhance genomic instability by inhibiting DNA repair [42], targeting protein neddylation could be an alternative approach to ignite tumor-specific immune responses against acquired resistance, through the recruitment and activation of innate immune cells.

Upon neddylation deletion, we observe up-regulated expression of interferon genes on cancer cells and recruitment of pro-inflammatory DC and myeloid cells. These benefits are lost when *Cgas* is knocked-out from neddylation-deficient tumors and compromised the superior response to PD1 blockade. Recently, Schiantarelli and colleagues showed that the diminished STING signaling in cancer cells led to a weakened innate immunity and that could drive acquired resistance to ICB therapy in patients [11]. It would be important to dissect the molecular network and functional hierarchy controlled by neddylation, cGAS/STING and JAK/STAT pathways during the formation of acquired resistance to immunotherapy.

To date, several direct agonists of the cGAS/STING pathway have been developed and tested in clinical studies [39]. Because systemic delivery of these agonists causes a broad immune activation with limited specificity to cancer-derived antigens, intra-tumoral stimulation of the pathway in cancer cells [43] or indirect activation of cGAS/STING by fine-tuning the availability of dsDNA in cancer cells [39] may be more beneficial. In our study, genetic deletion of cancer-intrinsic neddylation increases the chromatin-bound cGAS and bypasses the JAK/STAT pathway to unlock the interferon gene signatures *in vitro* and *in vivo*. Therefore, selective targeting of protein neddylation in solid tumors may offer an alternative therapeutic approach to elicit tumor-specific anti-tumor immune responses in synergy with ICB drugs.

## Materials and Methods

Details of all antibodies, reagents and oligonucleotide sequences can be found in **Supplementary Table 1, 2 and 3**.

### Study approvals

All mice were housed at the animal facility at the Department of Immunology, Genetics and Pathology in the Rudbeck laboratory at Uppsala University. All studies were approved by the Swedish Board of Agriculture at Jönköping, Sweden (Dnr: 5.8.18–06394/2020 and 5.8.18–05492/2025). Blood products from healthy donors were obtained in buffy coats from the Uppsala University Hospital. No ethical permission was required as donors were fully anonymous.

### Cell lines and gene deletion using CRISPR/Cas9

Human melanoma cell lines M420 and M464 were obtained from Dr. Antoni Ribas (UCLA, USA) [4] and maintained in Roswell Park Memorial Institute (RPMI) 1640 medium supplemented with 20% heat-inactivated Fetal Bovine Serum (FBS) and 1% penicillin-streptomycin (Thermo Fisher Scientific). Murine breast cancer cell line EO771 was kindly donated by Dr. Maria Ulvmar (Uppsala University, Sweden). Parental or genetically modified murine cell lines were maintained in Iscove’s Modified Dulbecco’s medium (IMDM, Thermo Fisher Scientific) supplemented with 10% FBS and 1% penicillin-streptomycin. All cell lines were cultured at 37°C with 5% carbon dioxide and routinely checked for mycoplasma infection (MycoAlert, Lonza). Individual gene deletion was performed using the Neon transfection system (Thermo Scientific) as previously described [21]. Briefly, a ribonucleoprotein complex (RNP) containing the two-part guide RNA of interest (CRISPR RNA (crRNA, IDT) + trans-activating CRISPR RNA (tracrRNA, Cas9, and carrier DNA ( all from IDT) were introduced into 3×10^5^ cancer cells by electroporation. A negative control without the crRNA was performed for each electroporation. To achieve a complete gene deletion, sequential rounds of electroporation were performed on cells. The complete list of crRNAs is included in Supplementary Table 1. The Incucyte zoom live imaging system (Sartorius) was used to determine the proliferation of cancer cells in vitro. In brief, cells were seeded in a 96-well flat bottom plate and transferred to the incucyte, where images were taken every 6 hours to assess confluency.

### In vitro treatment of cancer cells

After allowing for cell line adherence overnight, inhibitors or cytokines at the indicated concentrations or DMSO were added to the plate. To assess cGAS stabilization, cancer cells were seeded in a 6-well plate and treated with 500 nM of pevonedistat or DMSO control. Cells were harvested after one hour to extract cellular and subcellular lysates for protein detection using western blotting. To determine response to interferon gamma (IFNγ), cancer cells were seeded in a 6-well plate with or without 50 ng/mL recombinant IFNγ (Peprotech). Cells were harvested within the first hour to extract cell lysates for the detection of protein phosphorylation using western blotting or a phospho-kinase array (R&D Systems). Phenotypical changes on cancer cells were determined using flow cytometry at 24 hours.

### Label-free proteomics and data analysis

M420, M464 or *UBA3* KO M464 cells were seeded in 10cm^3^ dishes and left to adhere overnight. On the following day, media was renewed and supplemented with 50 ng/mL of recombinant IFNγ or the equivalent volume of PBS. Cells were treated for 24h, and then harvested with a soft-tip scraper and kept on ice as a cell pellet. The samples were then lysed in 150 μL of a buffer containing 1% β-octyl glucopyranoside and 6M urea using a sonication probe for 30 seconds (3 mm probe, pulse 1 second, 40% amplitude). Samples were then incubated for 60 minutes at 4°C with mild agitation. The lysates were clarified by centrifugation for 10 minutes at 14000 g. The supernatant containing the extracted proteins was collected and further processed. Total protein concentration in the sample was measured using the DC Protein Assay and BSA as the standard. 35 μg of protein from each of the samples was further processed. Proteins were reduced, alkylated, and on-filter digested by trypsin using 3 kDa centrifugal spin filter (Millipore, Ireland). The collected filtrate was vacuum centrifuged to dryness using a SpeedVac system. Then, peptides were separated in reversed-phase on a C18-column with 150-minute gradient and electrosprayed on-line to a Q-Exactive Plus mass spectrometer (Thermo Finnigan). Tandem mass spectrometry was performed applying HCD.

Proteins were quantified from the RAW-data file using MaxQuant software. Proteins were identified by searching towards proteins from Homo sapiens proteome extracted from Uniprot in February 2020 and May 2025. The search parameters were set to Taxonomy: Homo Sapiens; Enzyme: Trypsin; Fixed modification: Carbamidomethyl (C); Variable modifications: Phosphorylation (STY), Oxidation (M), Deamidated (NQ). Proteins detected in at least two of the replicates per condition were kept for differential protein expression. Proteins detected in 3 or 4 replicates in one group but not detected in the other were set as unique. After protein quantification, LFQ intensity values were corrected for background and normalized via variance stabilizing transformation using DEP package in R [44]. A linear model combined with empirical Bayes statistics was used for the differential expression analysis. Differentially expressed proteins were established as having an absolute Log2FC equal or greater than 0.5, and an adjusted p value below 0.05. Hypergeometric tests were then performed separately for upregulated and downregulated proteins to discover differently enriched pathways using clusterProfiler [45]. To uncover protein-protein interactions, dysregulated proteins were queried on STRING database and further studied with Cytoscape 3.10.3 [46].

### Western blotting

Cell lysates were prepared from cell pellets using radioimmunoprecipitation assay (RIPA) lysis buffer (1 mM EGTA, 20 mM Tris, 150 mM NaCl, 1 mM EDTA, 1% NP-40, 1 mM NaF, 1 mM NaVO3, 1 mM sodium phosphate) supplemented with protease inhibitor cocktail (Thermo Scientific). Additionally, phosphatase inhibitor cocktail (Thermo Scientific) was used when blotting for phosphorylated proteins. After a 20-minute lysis on ice, cells were centrifuged at 17000 g for 10 minutes at 4°C. To assess protein localization within the cells, subcellular fractionation was performed with a Subcellular Protein Fractionation Kit (Thermo Scientific). Cells were first treated with 500nM pevonedistat or 0.005% DMSO control for 1 hour, and then harvested using a soft-tip scraper and immediately placed on ice. Then, lysates from the different subcellular locations were obtained following the protocol as per manufacturer’s instructions. Next, protein concentrations were determined via the Bicinchoninic Acid (BCA) Assay (Thermo Scientific), using known Bovine Serum Albumin concentrations as a standard. Chromatin-bound protein concentration was measured using the Lite Plus (Thermo Scientific). After supplementing the lysates with SDS loading dye, samples were heated up at 70°C for 10 minutes and separated by 4–12% SDS-PAGE gel (Invitrogen). Proteins were transferred to a nitrocellulose membrane (Invitrogen) by dry electrotransfer using an iBlot2 device (Invitrogen). Membranes were incubated for one hour with 5% non-fat SKIM milk powder (Invitrogen) or Everyblot Blocking Buffer (Bio-Rad). After washing with TBS-T buffer (TBS with Tween20 at 0.1%), membranes were incubated with diluted primary antibody overnight at 4°C with gentle rocking. On the following day, HRP-linked secondary antibody (Cell Signaling Technology) was added to the membranes for 1 hour at room temperature. Super Signal West Pico Plus or West Femto chemiluminescent substrate (Thermo Scientific) was used to detect the bands based on target protein abundance.

To detect multiple phosphorylated proteins upon IFNγ signaling (50 ng/mL, 30 minutes), lysates were blotted with the Proteome Profiler Human Phospho Kinase Array (R&D Systems) as per manufacturer’s instructions. All images were captured on an Amersham Imager 680 (GE Healthcare).

### Human primary immune cell isolation

Peripheral blood mononuclear cells (PBMC) were isolated from buffy coats from healthy donors (Uppsala University Hospital, Sweden) by density gradient centrifugation using SepMate tubes (Stem Cell Technologies). In brief, 15mL of Lymphoprep reagent (Stem Cell Technologies) was added to the tubes, followed by the addition of 35mL of blood. Tubes were then centrifuged at 1200g for 10 minutes. The PBMC fraction was collected and washed twice with phosphate-buffered saline (PBS, Thermo Fisher Scientific) before lysing for red blood cells using 5mL of red blood cell lysis buffer (RBC, Thermo Fisher Scientific). Cells were incubated for 10 minutes in the dark, and then washed with PBS. Further enrichment of lymphocyte or isolation of monocytes was performed using the EasySep CD14+ selection kit II (Stem Cell Technology) as per manufacturer instructions. The spare PBMC resulting from blood isolation were stored in −150°C freezer until further use.

### Tumor immune co-culture system

A total of 5×10^3^ to 1×10^4^ human M420/M464 cancer cells were seeded per well in a 96-well flat bottom plate and left to incubate overnight to allow cell adherence. On the following day, lymphocytes were labelled with 5nM CellTrace Violet dye (CTV, Thermo Fisher Scientific) by incubating them in the dark for 10 minutes, and then washing twice with PBS. Next, 3×10^5^ CTV-labelled lymphocytes were added per well, in the presence of either nivolumab (Bristol-Myers Squibb) or durvalumab (AstraZeneca) at a final concentration of 10 μg/mL. In some experiments, neddylation inhibitor pevonedistat (MedChemExpress) or DMSO (0.005%) was added to the plate at the indicated concentrations on day 3 after the start of the co-culture. After a total of 6-day incubation, soluble IFNγ (BioLegend) and Granzyme B (MabTech) in culture supernatants were quantified by ELISA according to the manufacturer instructions. ELISA results were quantified by reading absorbance values at 450 and 570 nm on a CLARIOstar Plus instrument (BMG Labtech). Cells were harvested on the same day and immune cell proliferation and activation were measured using flow cytometry.

### Genome-wide CRISPR screens to identify resistance genes in M464 cells.

In order to uncover the cancer-intrinsic resistance network to nivolumab in *JAK2*-deficient M464 cells, genome-wide CRISPR screens were performed in TICS according to our previously published procedures [21, 22]. First, a stable Cas9-expressing M464 cell line was generated by transduction with a lentivirus pLenti-Cas9-T2A-Blast-BFP derived from lenti-dCAS9-VP64_Blast (a gift from Feng Zhang, Addgene). After blasticidin selection, cells were sorted (Sony SH800) sequentially to generate a stable BFP-expressing population with over 85% BFP+ cells. Brunello library was synthesized, packaged, and transduced to M464-Cas9 cells using the same protocol described by Papakyriacou et al. [21]. Cells were selected with puromycin at 2 μg/mL from day 2 to day 6 post transduction. For the screen with donor 1, cells were kept in culture from day 6 to day 10. For the screen with donor 2, frozen cells from day 6 post-transduction were thawed and grown until day 10. On day 10, cells were seeded in T175 at 3 million cells per flask, with 40 million cells per condition in total to ensure library coverage.

On the following day, healthy primary human lymphocytes were isolated as described above and evenly seeded into each flask. Two independent screens were performed using lymphocytes from independent blood donors at E:T ratios of 5.3:1 and 5.2:1 for donor 1 and 2 respectively. After 6 days of co-culture, floating cells were gently washed away and genomic DNA was isolated from cancer cells using the QIAmp DNA Blood Maxi kit (Qiagen). Guide cassettes were amplified by PCR [47] using the modified primers PCR2_fw acactctttccctacacgacgctcttccgatctcttgtggaaaggacgaaacac and PCR3_fw aatgatacggcgaccaccgagatctacac [i5] acactctttccctacacgacgctct. Amplicons were then sequenced on Illumina NovaSeq, reading 20 cycles with custom primer CGATCTCTTGTGGAAAGGACGAAACACCG; 10 cycles index read i7 to read the UMI, and six cycles index read i5 for the sample barcode. Media from each flask was sampled to measure levels of soluble IFNγ or Granzyme B using ELISA. The MAGeCKFlute R package [48] was used to output depleted pathways from Reactome [49] and Gene Ontology [50, 51] databases. A threshold for gene hits was set at mean −/+ 3 standard deviations from the mean score. Genes were selected and overlapped between both screens.

### Tumor growth in immune competent mice

Female C57BL/6NTac mice between 6 to 10 weeks old were purchased from Taconic and housed in a barrier facility at Rudbeck Laboratory with humidity ranging 45–65% and an average temperature of 23°C. The dark/light cycle was fixed to 12 hours. Parental or genetically modified EO771 cells (6 × 10^5^ cells per mouse) were injected subcutaneously in 100 μL of serum-free IMDM on the right flank. Tumor-bearing mice were treated intraperitoneally with 50 μg or 100 μg of anti-PD1 as specified (clone RMP1–14, BioXcell or AssayGenie) or a Rat isotype IgG2a control (clone 2A3, BioXCell; clone 1–1, AssayGenie) on day 7, 10 and 13 post tumor injection. Mice were checked 2–3 per week and tumor volumes were calculated using the formula V = length*width^2^/2. Tumor endpoint was considered after the volume exceeded 1 cm^3^. Mice were euthanized at the study endpoint and tumors and spleens were harvested. Single cells from tumor tissues were isolated using the GentleMACS instrument and the Murine Tumor Dissociation Kit (Miltenyi Biotech) as per manufacturer’s instructions. Single cells from spleens were obtained by mechanically dissociating the tissue and passing the cells through a 40μm cell strainer. Cells were then resuspended in 2mL of red blood lysis buffer for 3 minutes on ice, followed by washing in PBS.

### Flow cytometry

Multi-color flow cytometry was used to quantify protein expression on cancer cells upon genetic deletion and/or treatment, co-cultures as well as single cells generated from tumor-bearing mice. In brief, cells were seeded in a 96-well v-bottom plate and washed once with 200 μL PBS. Next, cells were resuspended in 20 μL of PBS containing fixable blue or aqua live/dead dye (Invitrogen) and a Fc receptor blocker (Thermo Fisher Scientific) and incubated for 20 minutes at 4°C. Then, cells were washed once with PBS and resuspended in 20 μL of PBS containing antibodies targeting surface markers. After a 25-minute incubation at 4°C, cells were washed twice and then resuspended in 150 μL of PBS for analysis. To detect intracellular proteins, cells were fixed and permeabilized using the CytoFix/Perm buffer set (BD Biosciences) or Cyto-Fast^™^ Fix/Perm kit (BioLegend), followed by washing and up to a 40-minute incubation with fluorochrome-conjugated antibodies at room temperature. Then, cells were washed twice with PBS and lastly resuspended in 150 μL of PBS for analysis.

All samples were analyzed using a CytoFLEX LX or S (Beckman coulter), as well as a LSR Fortessa (BD Biosciences). Data was then analyzed with FlowJo software V10.

### Fluorescence-Activated Cell Sorting (FACS)

FACS was used to isolate cell population based on protein surface expression. Briefly, cells were stained with aqua live/dead dye (1:200) with 100 μL every 1×10^6^ cells. EO771 cells were sorted based on B2m and H2-K/D expression after *B2m* CRISPR/Cas9 genetic deletion. After two washes with PBS, cells were resuspended in 1 mL PBS and sorted using the Aria III sorter (BD Biosciences) or CytoFLEX SRT (Beckman Coulter).

### Single-cell RNA-Seq from murine tumors

Cells from murine tumors were processed using GentleMACS as described above. After red blood cell lysis, tumor cells were stained with aqua live/dead dye (1:150) and incubated at 4°C for 30 minutes. After a wash with PBS, cells were resuspended in 1mL PBS and 3–5×10^5^ alive cells were sorted for each sample. Then, cells were fixed using the 10x Genomics kit as per manufacturer instructions. In brief, cells were centrifuged after sorting and the cell pellets were fixed for 20 hours at 4°C using the Fix & Perm buffer containing 4% formaldehyde. Then, cells were centrifuged (1000g, 10 minutes) and quenching buffer was added to the pellets. Enhancer was then thawed at 65°C for 10 minutes and immediately added to the samples. Finally, 50% glycerol was added to each sample for a final concentration of 10%, and cells were stored at −80°C before being sequenced.

Gene expression of fixed samples was evaluated following Chromium Fixed RNA Profiling (Gene Expression FLEX). In brief, samples were hybridized with barcoded probes and the sequencing library was generated following the User Guide (CG000527). Sample concentrations were evaluated with the automated cell counter LunaFX7, before the hybridization step, which was performed with the 16-barcoded probe kit. Next, samples were incubated at 42 degrees overnight for 22.5 hours. After hybridization, samples were pooled and cells were washed and filtered following the User Guide. The cell concentration of the multiplexed pool was determined before loading to the Chromium X chip, and targeted cell recovery was aimed at 1.28×10^5^ cells. The library was sequenced on the Illumina NovaSeq X Plus system, according to manufacturer’s menu. FASTQ files were processed with Cell Ranger v8.0.1 (10X Genomics) using the Mouse Reference Genome Mm10 2020 A.

### Analysis of scRNA-seq datasets

Single cell RNAseq expression matrix, barcode and metadata files were integrated and analyzed using Seurat (v5.1.0) [52]. Low quality cells were excluded from further analysis based on common quality control thresholds (number of UMIs, mitochondrial gene ratio, and number of genes detected <5th percentile or above 95th percentile; hemoglobin gene and heat shock protein ratio > 95th percentile; cell complexity < 5th percentile). The cell complexity score wass calculated for each cell using the formula:

$$\text{Cell}\;\text{complexity}=\frac{{\text{log}}_{10}(\text{number}\;\text{of}\;\text{gene}\;\text{detected})}{{\text{log}}_{10}(\text{number}\;\text{of}\;\text{UMIs})}$$

SCTransform (v 0.4.1) [53] was applied to normalize expression counts. Cell cycle scores were calculated with Seurat CellCycleScoring() function, as well as cell cycle differences (S cycle Score - G2M cycle Score). A total of 3000 variable genes were identified and principal component analysis (PCA) was applied to the dataset to reduce dimensionality after regressing for the number of features, cell cycle differences, percentage of mitochondrial genes, hemoglobin genes, and heat shock protein genes. Depending on the biological variance of the object for sub-clustering, the 20–50 most informative principal components were used for Uniform Manifold Approximation and Projection (UMAP) for dimension reduction. After the removal of doublets with *DoubletFinder()* (v2.0.6, https://github.com/blaserlab/doubletfinder), cell types and specific lineages were determined based on the expression of canonical cell markers. Pseudotime trajectory analysis of dendritic cell subsets was performed using Slingshot [54].

InferCNV (v1.20.0, https://github.com/broadinstitute/inferCNV) was utilized to calculate cellular copy number variation and assess epithelial cell malignancy, with randomly down-sampled immune cells used as reference. Differential gene expression was calculated with Seurat FindMarkers function, using the default Wilcoxon Rank Sum test and Bonferroni p value adjustment. Significant DEGs (absolute log2 fold change > 0.25, adjusted p-value < 0.05) were ranked based on log2 fold change and input into GSEA v4.3.2 [55, 56] for pathway enrichment analysis. Multi-nichnet [57] (https://github.com/saeyslab/multinichenetr) was used for cell-cell interaction analysis. Genes expressed in less than 5% of cells in a sample were excluded from that sample and considered not detected. Interactions that were either only detected in one group or had a 20% higher prioritization score compared to the other were labeled as upregulated.

### Single-cell RNA-Seq from publicly available human datasets

Processed single-cell RNA-Seq data [27] was downloaded from the Zenodo database using the accession code 15603513. Patient clinical data and immune infiltration calculated in the original study were downloaded from the supplementary material provided by the authors. RDS file containing all 189K cells after quality control and cluster assignment was uploaded into R as a Seurat object. AUCell package [58] was used to determine the single-cell enrichment of pathways from Reactome [49] and Hallmarks [59] databases containing the following terms: Interferon, IFN, or neddylation.

Patients were then separated based on the neddylation levels between high and low based on average enrichment of neddylation pathway within cancer cells. Patients were classified as low neddylation if the average value of REACTOME_NEDDYLATION pathway enrichment in cancer cells was below the 33^rd^ percentile, and high if it was above the 66^th^ percentile. Cancer cell pathway enrichment was performed at a per-cell basis between neddylation low and high patients using fgsea [60]. Wilcoxon test was then performed to evaluate mean differences of clinical and immune abundance variables between the two groups.

### Statistical analysis

All flow cytometry data was analyzed with FlowJo v10. The appropriate statistical tests for *in vitro* and *in vivo* experiments were performed in GraphPad Prism 10. R 4.3.3 was used to determine statistical significance in omics data. Wilcoxon rank-sum test or Wilcoxon matched-pairs signed rank test were performed to assess differences between two groups with unpaired or paired samples, respectively. Two-way ANOVA tests were used for comparing multiple experimental groups. All tests were two-tailed, and P value adjustment was performed with Bonferroni correction, with statistical significance determined below a threshold of 0.05. For survival analyses, the log-rank Mantel-Cox test was used to determine statistical significance.

## Supplementary Files

This is a list of supplementary files associated with this preprint. Click to download.

- RubiesBedosFigureS1S10.pptx
- RubiesBedosSupplemental.docx

## References

[1] van Weverwijk A. and de Visser K.E., Mechanisms driving the immunoregulatory function of cancer cells. Nat Rev Cancer, 2023. 23(4): p. 193–215. 36717668 10.1038/s41568-022-00544-4

[2] Sharma P., , Primary, Adaptive, and Acquired Resistance to Cancer Immunotherapy. Cell, 2017. 168(4): p. 707–723. 28187290 10.1016/j.cell.2017.01.017 PMC5391692

[3] Ploegh H.L., Orr H.T., and Strominger J.L., Major histocompatibility antigens: the human (HLA-A, -B, -C) and murine (H-2K, H-2D) class I molecules. Cell, 1981. 24(2): p. 287–99. 7016338 10.1016/0092-8674(81)90318-4

[4] Zaretsky J.M., , Mutations Associated with Acquired Resistance to PD-1 Blockade in Melanoma. N Engl J Med, 2016. 375(9): p. 819–29. 27433843 10.1056/NEJMoa1604958 PMC5007206

[5] Sade-Feldman M., , Resistance to checkpoint blockade therapy through inactivation of antigen presentation. Nat Commun, 2017. 8(1): p. 1136. 29070816 10.1038/s41467-017-01062-w PMC5656607

[6] Gettinger S., , Impaired HLA Class I Antigen Processing and Presentation as a Mechanism of Acquired Resistance to Immune Checkpoint Inhibitors in Lung Cancer. Cancer Discov, 2017. 7(12): p. 1420–1435. 29025772 10.1158/2159-8290.CD-17-0593 PMC5718941

[7] Torrejon D.Y., , Antitumor Immune Responses in B2M-Deficient Cancers. Cancer Immunol Res, 2023. 11(12): p. 1642–1655. 37801341 10.1158/2326-6066.CIR-23-0139 PMC10842455

[8] Shin D.S., , Primary Resistance to PD-1 Blockade Mediated by JAK1/2 Mutations. Cancer Discov, 2017. 7(2): p. 188–201. 27903500 10.1158/2159-8290.CD-16-1223 PMC5296316

[9] Sucker A., , Acquired IFNgamma resistance impairs anti-tumor immunity and gives rise to T-cellresistant melanoma lesions. Nat Commun, 2017. 8: p. 15440. 28561041 10.1038/ncomms15440 PMC5460020

[10] Gao J., , Loss of IFN-gamma Pathway Genes in Tumor Cells as a Mechanism of Resistance to Anti-CTLA-4 Therapy. Cell, 2016. 167(2): p. 397–404 e9. 27667683 10.1016/j.cell.2016.08.069 PMC5088716

[11] Schiantarelli J., , Genomic mediators of acquired resistance to immunotherapy in metastatic melanoma. Cancer Cell, 2025. 43(2): p. 308–316 e6. 39933900 10.1016/j.ccell.2025.01.009 PMC12232206

[12] Roehle K., , cIAP1/2 antagonism eliminates MHC class I-negative tumors through T cell-dependent reprogramming of mononuclear phagocytes. Sci Transl Med, 2021. 13(594). 10.1126/scitranslmed.abf5058 PMC8406785 34011631

[13] Torrejon D.Y., , Overcoming Genetically Based Resistance Mechanisms to PD-1 Blockade. Cancer Discov, 2020. 10(8): p. 1140–1157. 32467343 10.1158/2159-8290.CD-19-1409 PMC7416458

[14] Williams J.B., , Tumor heterogeneity and clonal cooperation influence the immune selection of IFN-gamma-signaling mutant cancer cells. Nat Commun, 2020. 11(1): p. 602. 32001684 10.1038/s41467-020-14290-4 PMC6992737

[15] Kalbasi A., , Uncoupling interferon signaling and antigen presentation to overcome immunotherapy resistance due to JAK1 loss in melanoma. Sci Transl Med, 2020. 12(565). 10.1126/scitranslmed.abb0152 PMC8053376 33055240

[16] Kalbasi A. and Ribas A., Tumour-intrinsic resistance to immune checkpoint blockade. Nat Rev Immunol, 2020. 20(1): p. 25–39. 31570880 10.1038/s41577-019-0218-4 PMC8499690

[17] Li F., , Programmable bacteria synergize with PD-1 blockade to overcome cancer cell-intrinsic immune resistance mechanisms. Sci Immunol, 2024. 9(100): p. eadn9879. 39423284 10.1126/sciimmunol.adn9879 PMC11984541

[18] Manguso R.T., , In vivo CRISPR screening identifies Ptpn2 as a cancer immunotherapy target. Nature, 2017. 547(7664): p. 413–418. 28723893 10.1038/nature23270 PMC5924693

[19] Dubrot J., , In vivo CRISPR screens reveal the landscape of immune evasion pathways across cancer. Nat Immunol, 2022. 23(10): p. 1495–1506. 36151395 10.1038/s41590-022-01315-x

[20] Baumgartner C.K., , The PTPN2/PTPN1 inhibitor ABBV-CLS-484 unleashes potent anti-tumour immunity. Nature, 2023. 622(7984): p. 850–862. 37794185 10.1038/s41586-023-06575-7 PMC10599993

[21] Papakyriacou I., , Loss of NEDD8 in cancer cells causes vulnerability to immune checkpoint blockade in triple-negative breast cancer. Nat Commun, 2024. 15(1): p. 3581. 38678024 10.1038/s41467-024-47987-x PMC11055868

[22] Nagarajan D., , Epigenetic regulation of cell state by H2AFY governs immunogenicity in high-risk neuroblastoma. J Clin Invest, 2024. 134(21). 10.1172/JCI175310 PMC11527455 39255035

[23] Zhang S., , Protein neddylation and its role in health and diseases. Signal Transduct Target Ther, 2024. 9(1): p. 85. 38575611 10.1038/s41392-024-01800-9 PMC10995212

[24] McGrail D.J., , Proteome Instability Is a Therapeutic Vulnerability in Mismatch Repair-Deficient Cancer. Cancer Cell, 2020. 37(3): p. 371–386 e12. 32109374 10.1016/j.ccell.2020.01.011 PMC7337255

[25] Xu P., , The CRL5-SPSB3 ubiquitin ligase targets nuclear cGAS for degradation. Nature, 2024. 627(8005): p. 873–879. 38418882 10.1038/s41586-024-07112-w PMC10972748

[26] Plata-Gomez A.B., , Mitochondrial lipid metabolism in tumor immunosurveillance and evasion. Trends Immunol, 2025. 46(12): p. 766–778. 40962643 10.1016/j.it.2025.08.005

[27] Yang J., , Mature and migratory dendritic cells promote immune infiltration and response to anti-PD-1 checkpoint blockade in metastatic melanoma. Nat Commun, 2025. 16(1): p. 8151. 40890106 10.1038/s41467-025-62878-5 PMC12402436

[28] Zhou S., , Neddylation inhibition upregulates PD-L1 expression and enhances the efficacy of immune checkpoint blockade in glioblastoma. Int J Cancer, 2019. 145(3): p. 763–774. 31044422 10.1002/ijc.32379

[29] Chen J.H., , Human lung cancer harbors spatially organized stem-immunity hubs associated with response to immunotherapy. Nat Immunol, 2024. 25(4): p. 644–658. 38503922 10.1038/s41590-024-01792-2 PMC12096941

[30] Barry K.C., , A natural killer-dendritic cell axis defines checkpoint therapy-responsive tumor microenvironments. Nat Med, 2018. 24(8): p. 1178–1191. 29942093 10.1038/s41591-018-0085-8 PMC6475503

[31] Bottcher J.P., , NK Cells Stimulate Recruitment of cDC1 into the Tumor Microenvironment Promoting Cancer Immune Control. Cell, 2018. 172(5): p. 1022–1037 e14. 29429633 10.1016/j.cell.2018.01.004 PMC5847168

[32] Oh S.A., , PD-L1 expression by dendritic cells is a key regulator of T-cell immunity in cancer. Nat Cancer, 2020. 1(7): p. 681–691. 35122038 10.1038/s43018-020-0075-x

[33] Peng Q., , PD-L1 on dendritic cells attenuates T cell activation and regulates response to immune checkpoint blockade. Nat Commun, 2020. 11(1): p. 4835. 32973173 10.1038/s41467-020-18570-x PMC7518441

[34] Mayoux M., , Dendritic cells dictate responses to PD-L1 blockade cancer immunotherapy. Sci Transl Med, 2020. 12(534). 10.1126/scitranslmed.aav7431 32161104

[35] Maier B., , A conserved dendritic-cell regulatory program limits antitumour immunity. Nature, 2020. 580(7802): p. 257–262. 32269339 10.1038/s41586-020-2134-y PMC7787191

[36] Hammerich L., , Systemic clinical tumor regressions and potentiation of PD1 blockade with in situ vaccination. Nat Med, 2019. 25(5): p. 814–824. 30962585 10.1038/s41591-019-0410-x

[37] Oh J., , Spatial analysis identifies DC niches as predictors of pembrolizumab therapy in head and neck squamous cell cancer. Cell Rep Med, 2025. 6(5): p. 102100. 40311615 10.1016/j.xcrm.2025.102100 PMC12147904

[38] Lee C.Y.C., , Tumour-retained activated CCR7(+) dendritic cells are heterogeneous and regulate local anti-tumour cytolytic activity. Nat Commun, 2024. 15(1): p. 682. 38267413 10.1038/s41467-024-44787-1 PMC10808534

[39] Fahey C.G., , Targeting STING to generate therapeutic anti-tumor immunity. Cancer Cell, 2025. 10.1016/j.ccell.2025.12.002 PMC12779296 41448179

[40] Falahat R., , Epigenetic reprogramming of tumor cell-intrinsic STING function sculpts antigenicity and T cell recognition of melanoma. Proc Natl Acad Sci U S A, 2021. 118(15). 10.1073/pnas.2013598118 PMC8053941 33827917

[41] Cryer A.M., , Restoration of cGAS in cancer cells promotes antitumor immunity via transfer of cancer cell-generated cGAMP. Proc Natl Acad Sci U S A, 2025. 122(45): p. e2409556122. 41183190 10.1073/pnas.2409556122 PMC12625989

[42] Jiang H., , Chromatin-bound cGAS is an inhibitor of DNA repair and hence accelerates genome destabilization and cell death. EMBO J, 2019. 38(21): p. e102718. 31544964 10.15252/embj.2019102718 PMC6826206

[43] Garland K.M., Sheehy T.L., and Wilson J.T., Chemical and Biomolecular Strategies for STING Pathway Activation in Cancer Immunotherapy. Chem Rev, 2022. 122(6): p. 5977–6039. 35107989 10.1021/acs.chemrev.1c00750 PMC8994686

[44] Zhang X., , Proteome-wide identification of ubiquitin interactions using UbIA-MS. Nat Protoc, 2018. 13(3): p. 530–550. 29446774 10.1038/nprot.2017.147

[45] Yu G., , clusterProfiler: an R package for comparing biological themes among gene clusters. OMICS, 2012. 16(5): p. 284–7. 22455463 10.1089/omi.2011.0118 PMC3339379

[46] Shannon P., , Cytoscape: a software environment for integrated models of biomolecular interaction networks. Genome Res, 2003. 13(11): p. 2498–504. 14597658 10.1101/gr.1239303 PMC403769

[47] Schmierer B., , CRISPR/Cas9 screening using unique molecular identifiers. Mol Syst Biol, 2017. 13(10): p. 945. 28993443 10.15252/msb.20177834 PMC5658704

[48] Wang B., , Integrative analysis of pooled CRISPR genetic screens using MAGeCKFlute. Nat Protoc, 2019. 14(3): p. 756–780. 30710114 10.1038/s41596-018-0113-7 PMC6862721

[49] Milacic M., , The Reactome Pathway Knowledgebase 2024. Nucleic Acids Res, 2024. 52(D1): p. D672–D678. 37941124 10.1093/nar/gkad1025 PMC10767911

[50] Ashburner M., , Gene ontology: tool for the unification of biology. The Gene Ontology Consortium. Nat Genet, 2000. 25(1): p. 25–9. 10802651 10.1038/75556 PMC3037419

[51] Gene Ontology C., The Gene Ontology knowledgebase in 2026. Nucleic Acids Res, 2026. 54(D1): p. D1779–D1792. 41413728 10.1093/nar/gkaf1292 PMC12807639

[52] Hao Y., , Dictionary learning for integrative, multimodal and scalable single-cell analysis. Nat Biotechnol, 2024. 42(2): p. 293–304. 37231261 10.1038/s41587-023-01767-y PMC10928517

[53] Hafemeister C. and Satija R., Normalization and variance stabilization of single-cell RNA-seq data using regularized negative binomial regression. Genome Biol, 2019. 20(1): p. 296. 31870423 10.1186/s13059-019-1874-1 PMC6927181

[54] Street K., , Slingshot: cell lineage and pseudotime inference for single-cell transcriptomics. BMC Genomics, 2018. 19(1): p. 477. 29914354 10.1186/s12864-018-4772-0 PMC6007078

[55] Subramanian A., , Gene set enrichment analysis: a knowledge-based approach for interpreting genome-wide expression profiles. Proc Natl Acad Sci U S A, 2005. 102(43): p. 15545–50. 16199517 10.1073/pnas.0506580102 PMC1239896

[56] Mootha V.K., , PGC-1alpha-responsive genes involved in oxidative phosphorylation are coordinately downregulated in human diabetes. Nat Genet, 2003. 34(3): p. 267–73. 12808457 10.1038/ng1180

[57] Browaeys R., Saelens W., and Saeys Y., NicheNet: modeling intercellular communication by linking ligands to target genes. Nat Methods, 2020. 17(2): p. 159–162. 31819264 10.1038/s41592-019-0667-5

[58] Aibar S., , SCENIC: single-cell regulatory network inference and clustering. Nat Methods, 2017. 14(11): p. 1083–1086. 28991892 10.1038/nmeth.4463 PMC5937676

[59] Liberzon A., , The Molecular Signatures Database (MSigDB) hallmark gene set collection. Cell Syst, 2015. 1(6): p. 417–425. 26771021 10.1016/j.cels.2015.12.004 PMC4707969

[60] Korotkevich G., , Fast gene set enrichment analysis. bioRxiv, 2021: p. 060012.

